# Phytochemical, In Silico, In Vitro, and In Vivo Research on *Piptadeniastrum africanum* (Fabaceae) Unveiling Anti‐Stereotypic, Anxiolytic, and Analgesic Effects in a Sodium Valproate‐Induced Autistic Disorders Model

**DOI:** 10.1002/brb3.70408

**Published:** 2025-03-13

**Authors:** Ambani Omgba Jeanne Julie, Ngouateu Omer Bébé, Mengue Ngadena Yolande Sandrine, Owona Pascal Emmanuel, Kandeda Kavaye Antoine, Ambamba Akamba Bruno Dupon, Nongni Piebeng Quentin Cicilien, Ngang Nguema Franck Emmanuel, Ngondi Judith Laure, Bilanda Danielle Claude, Dzeufiet Djomeni Paul Désiré

**Affiliations:** ^1^ Department of Animal Biology and Physiology, Laboratory of Animal Physiology, Faculty of Science University of Yaoundé 1 Yaoundé Cameroon; ^2^ Neurosciences axis, Laboratory of Development and Maldevelopment, Department of Psychology, Faculty of Arts, Letters, and Social Science University of Yaoundé 1 Yaoundé Cameroon; ^3^ Department of Biochemistry, Faculty of Science University of Yaoundé 1 Yaoundé Cameroon; ^4^ Center of Nutrition and Functional Foods Yaoundé Cameroon

**Keywords:** analgesic, anxiolytic, autistic disorders, *Piptadeniastrum africanum*, sodium valproate

## Abstract

**Objective:**

Individuals with autistic spectrum disorders (ASD) primarily exhibit deficits in communication and social interaction, along with repetitive behaviors and restricted interests. This disorder is often associated with anxiety, nociceptive disorders, and pain. While medical treatment generally focuses on treating the symptoms rather than addressing the underlying causes, traditional medicine is sometimes used as an alternative. *Piptadeniastrum africanum* is used in Cameroonian medicinal folks to treat cognitive disorders. However, its effects and mechanisms of action regarding the inhibition of ASD‐like symptoms remain unclear. The primary goal of the present study was to evaluate the anxiolytic and analgesic effects of the water extract of *P. africanum* on autistic triad induced in rats by sodium valproate.

**Material and Methods:**

The study investigated the secondary metabolites in *P. africanum* extract using UHPLC‐MS. DPPH, ABTS, and FRAP tests were performed to assess the extract's ability to neutralize free radicals. Molecular docking was utilized to evaluate the extract's binding to various receptors. For the experimental study, 33 pregnant female rats were divided into two groups after pregnancy was confirmed. One group was given distilled water orally at 10 mL/kg, while the other group received sodium valproate at 800 mg/kg on gestation days 11, 12, and 13. When the male offspring reached 3 weeks old, they were evaluated for anxiety, social interaction, and pain sensitivity, with those displaying any disorders selected for further study. The remaining rats were split into six groups of five and treated with either a vehicle, bumetanide, or *P. africanum* extract at 190 and 760 mg/kg. Behavioral assessments focusing on sociability, anxiety, and pain sensitivity were conducted on days 28 and 37 after weaning. In the end, biochemical markers related to GABA metabolism, serotonin levels, and oxidative status were analyzed in the cerebellum, prefrontal cortex, hippocampus, and amygdala alongside histopathological analyses in the brain.

**Results:**

UHPLC‐MS allows us to identify several compounds. They bind to H3R (7F61) and HDAC2 through conventional hydrogen bonding. Findings showed that prenatal administration of sodium valproate induced in male offspring a deficit in social interaction (*p* < 0.001), anxiety disorders (*p* < 0.001), hypersensitivity to pain (*p* < 0.001), increased GABA and serotonin concentration (*p* < 0.001), disturbed oxidative status (*p* < 0.001), and neuronal loss (*p* < 0.001) as well as neuronal disorganization in the hippocampus, cerebellum and amygdala in young rats compared to neurotypical animals. *P. africanum* extract at doses used, like bumetanide, corrected these disorders and protected against neuronal loss. These results suggest that the extract has anxiolytic and anti‐nociceptive effects. It has been found that the positive effects can be achieved by restoring GABAergic and serotonergic neurotransmission, coupled with antioxidant and neuromodulatory activity.

**Conclusion:**

The current findings support that *P. africanum* induces anxiolytic and analgesic effects in a sodium valproate‐induced autistic disorders model.

## Graphical abstract

The aim of the present study was to evaluate the anxiolytic and analgesic effects of the water extract of P. africanum on autistic triad induced in rats with sodium valproate. Pregnant female rats were given sodium valproate at 800 mg/kg during gestation. Male offspring were evaluated for anxiety, social interaction, and pain sensitivity, and treated with P. africanum extract. Molecular docking was utilized to evaluate how well the extract binds to various receptors. P. africanum compounds bind to H3R (7F61) and HDAC2 through conventional hydrogen bonding. P. africanum has anxiolytic and anti‐nociceptive effects by restoring GABAergic and serotonergic neurotransmission, coupled with antioxidant and neuromodulatory activity.

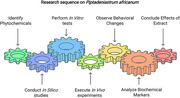

**Abbreviations**
5‐HT: serotonin
ASD: autism spectrum disorder
CA1: Cornu ammonis 1
CA3: Cornu ammonis 3
CNS: central nervous system
GABA: Gamma‐Aminobutyric acid
GABA‐T: GABA‐transaminase
GSH: reduced glutathione
L‐GAD: glutamate decarboxylase
MDA: malondialdehyde
NKCC1: Na^+^‐K^+^‐Cl^−^ cotransporter
NT: neurotypical
*P. africanum*
: *Piptadeniastrum africanum*
SOD: superoxide dismutase
VPA: sodium valproate

## 1 Introduction

Autistic spectrum disorder (ASD) is a neurodevelopmental disorder that affects almost 1 in 100 children globally, with a ratio of 4 boys to 1 girl (WHO 2022). The traumas and social consequences caused by ASD are becoming a real public health problem. According to the Ministry of Public Health, almost 3000 children are born autistic in Cameroon each year (Mbassi et al. 2017). Clinically, ASD is mainly characterized by deficits in communication and social interaction, as well as repetitive behaviors and restricted centers of interest (Desaunay et al. 2014). ASD included Rett's syndrome, childhood disintegrative disorders, atypic, infantile, and high‐potential autism. Several theories exist to explain the origins of ASD (Ha et al. 2015). Two of these have been established based on observations from animal models. One theory concerns the deregulation of the excitation/inhibition balance in neural networks, while the other is based on neurons' extreme excitability and plasticity. ASD can be accompanied by several comorbidities, such as depression, gastric disorders, pain, epilepsy, and eye disorders, due to the mental and hormonal imbalances associated with this condition (Elsabbagh et al. 2012).

ASD is thought to arise from a mix of genetic and environmental influences, including exposure to certain substances like sodium valproate (VPA) during fetal development (Bossu and Roux 2019). Research using animal models has demonstrated that prenatal exposure to VPA effectively mimics key characteristics of ASD, showing face, conceptual, and predictive validity (Sharma et al. 2022). VPA is an anticonvulsant that is used to treat epilepsy during pregnancy, but high doses can cause congenital malformations. The behavioral and biochemical changes seen in rats exposed to VPA during neural tube closure (days 11–13 of pregnancy) are similar to those seen in ASD (Ukkirapandian et al. 2022). Although there is no known cure for ASD, drug treatments can help manage symptoms. Even though ASD is diverse, some common treatment targets are being discovered on a biological level. This may eventually lead to the development of urgently needed etiology‐driven treatments. Among others, nematin and agmatine can improve social behavior and reduce stereotypies, while oxytocin can help with anxiety (Pagan 2012). Furthermore, clonidine, naltrexone, lithium, mega vitamins, thyroid hormones, and antidepressants are also used as symptomatic treatment, but none of them has a significant effect. Bumetanide is a loop diuretic of the sulfonamide class that inhibits cation‐chloride co‐transporter. It can restore GABA inhibitory function and improve the autistic behavioral triad. However, it has several side effects, such as addiction, gastric disorders, muscular pain, and convulsions. Moreover, it is expensive and inaccessible in rural areas (Nicolini and Fahnestock 2018; Wang et al. 2021; Hiremath and Srinivas 2022).

Research has pointed to the histamine H3 receptor (H3R) as a potentially significant factor linked to autism ASD (Eissa et al. 2020). The H3R is a subtype of histamine receptors that plays a crucial role in regulating the release of neurotransmitters in the brain, including essential ones like 5‐HT and GABA (Szukiewicz 2024). These neurotransmitters have a profound impact on mood, cognition, and behavior, indicating that disruptions in H3R function may contribute to the atypical neurodevelopment seen in individuals with ASD. In addition to neurotransmitter activity, histone deacetylases (HDACs) are also emerging as key players in the epigenetic underpinnings of neurodevelopmental disorders, including ASD. The Autism Spectrum Disorders Working Group of The Psychiatric Genomics Consortium (2017) highlights that HDACs regulate gene expression by removing acetyl groups from histones, which can compact the chromatin structure and make it transcriptionally inactive. This epigenetic mechanism is vital, influencing how genes react to various developmental signals. Moreover, evidence indicates that heightened activity of HDAC2 may restrict the expression of genes essential for normal brain development. When HDAC2 becomes dysregulated, it can suppress these critical genes, potentially leading to behaviors and traits similar to those associated with ASD (Mbadiwe and Millis 2013). Specifically, the changes in gene expression influenced by HDAC2 might disrupt typical neurological processes and brain structure, significantly affecting social behaviors and interactions—core elements of ASD (Jiang et al. 2022). Therefore, delving into the relationship between histamine receptors like H3R and the role of HDACs may offer valuable insights into the mechanisms underlying autism spectrum disorders.

Recently, medicinal plants have gained popularity as a dependable source of preparing new drugs to treat various ailments. These plants are readily available, cost‐effective, and have fewer side effects than chemical drugs. They have shown promising results in treating autism, providing hope for people with ASD. It is worth noting that around 40% of pharmaceutical products are derived from natural products, and some of the leading medicines are based on traditional medicine. Traditional knowledge and medicine have contributed to groundbreaking medical discoveries, and herbal medicine has successfully treated specific pathologies. Medicinal plants could turn out to be an affordable and accessible source of relief for people with ASD (WHO 2023. *Piptadeniastrum africanum* is a forest plant that grows in lowland evergreen and semi‐deciduous forests in West and Central Africa. The plant's bark is traditionally used for medicinal purposes to treat gastric pain, mental disorders, and sinusitis (Ladoh et al. 2016). Recent research on a methanolic extract of *P. africanum* bark showed promising antioxidant and inhibitory activity against certain enzymes involved in type 2 diabetes and Alzheimer's disease (Sinan et al. 2020). Although the plant is known to treat several neurological disorders, there is limited data on its potential benefits for ASD symptoms. Therefore, this study investigated whether the aqueous extract of *P. africanum* could mitigate ASD‐like symptoms in a sodium valproate‐induced rat model. Specifically, the plant's anti‐stereotypic, anxiolytic, and analgesic effects have been evaluated. The hope is that this research will provide further insights into exploring the use of *P. africanum* as a potential multitarget treatment for ASD‐related symptoms.

## 2 Material and Methods

All methods were conducted in compliance with relevant guidelines, regulations, and legislation.

### 2.1 Chemical Substances

The chemical substances used in this study came from Hexal AG Industries tr.25 83607 (Holzkirchen Germany) for sodium valproate and from Laboratoires Leo 39 Route de Chartres 28501 Vernouillet cedex for Bumetanide marketed under the name Burinex 1.0 mg. The following chemicals were obtained from Siglma Ald Louis in MO, USA: methanol, orthophenantroline, iron sulfate (FeSO_4_), 2,2‐diphenyl‐I‐picryhydrazyl (DPPH), 2,2‐azinobis(3‐ethylbenzothiazoline‐6‐sulfonic acid) (ABTS), sodium chloride (NaCl), potassium permanganate (KMnO_4_) or potassium persulfate (K_2_S_2_O_8_), rutin, gallic acid, caffeic acid, catechin, and quillaja.

Sodium valproate (VPA) was prepared at 80 mg/mL by solubilization into distilled water and administered to animals at 800 mg/kg. Similarly, Bumetanide was prepared at 0.4 mg/mL and administered to the animals at 4 mg/kg.

### 2.2 Plant Material

The bark of *P. africanum* was collected in October 2021 from the Centre Region of Cameroon during the rainy season in the morning. The plant was identified in the National Herbarium of Cameroon by comparing it with sample number 19102/SRF/cam. The harvested barks were washed, and the sapwood (the white part of the bark) was removed with a knife per the traditional healer's instructions. The 13.5 g of sapwood obtained was macerated in 40 mL of distilled water for 6 h. The mixture was then filtered using Whatman N°3 paper, and the water was evaporated in an oven at 45°C. This process yielded 0.61 g of dry aqueous extract of *P. africanum*, equivalent to a yield of 4.52%.

#### 2.2.1 Phytochemical Profiling

Quantitative tests have been used for the phytochemical analysis of the aqueous extract of the bark sapwood of *P. africanum*. The reaction of quercetin with aluminum chloride and sodium acetate was used to determine the flavonoid content of the extract according to the method described by Zhishen et al. (1999). The spectrophotometry technique using the Folin‐Ciocalteu reagent described by Kupina et al. ()^2018^ made it possible to assess the level of total polyphenols present in the extract. The determination of the tannins was carried out using the method described by Broadhurst and Jones (1978) using acidified vanillin and tannic acid as standard.

#### 2.2.2 UHPLC‐MS Instrumentation

High‐resolution mass spectra (HRMS/MS) were obtained with a QTOF Spectrometer (Bruker, Germany) with a HESI source. The spectrometer was operated in negative mode (mass range: 100–1500, with a scan rate of 1.00 Hz) with automatic gain control to provide high‐accuracy mass measurements within 0.40 ppm deviation using Na Formate as a calibrant. The following parameters were used for experiments: spray voltage of 3.5 kV and capillary temperature of 200°C. Nitrogen was used as sheath gas (10 L/min). The spectrometer was attached to an Ultimate 3000 (Thermo Fisher, USA) UHPLC system consisting of an LC‐pump, Diode Array Detector (DAD) (λ: 190–600 nm), auto sampler (injection volume (5 µL) and column oven (35°C). The separations were performed using a Synergy MAX‐RP 100A (50 × 2 mm, 2.5 μ particle size) with an H_2_O (+0.1 % HCOOH) (A) / Acetonitrile (+0.1 % HCOOH) (B) gradient (flow rate 500 µL/min, injection volume 5 µL). Samples were analyzed using a gradient program as follows: 95% A isocratic for 1.5 min, linear gradient to 100% B over 6 min, after 100% B isocratic for 2 min, the system returned to its initial condition (90% A) within 1 min, and was equilibrated for 1 min.

### 2.3 Molecular Docking

The docking study was completed with MEO 2014 (Molecular Operating Environment [MEO]). For docking studies, (1) water was removed from the protein, (2) hydrogen atoms (H) with their standard geometry were added to the structure, and then the broken bonds were reconnected and the potential fixed. (3) For the search of large sites in the enzyme structure, the MEO Alpha Site Finder was used, and dummy atoms were generated from the resulting alpha spheres. (4) Analysis of the ligand interaction with the active sites of amino acids. The ligand binding affinity was evaluated using the scoring function dock function (S, Kcal/mol) created by the MOE 2014 software (Al‐Karmalawy et al. 2021). Active ligands with the highest docking score have the most negative values.

### 2.4 In Vitro Assays of Antioxidant Activities of *P. africanum*

In experiments, the percentage of inhibition was determined by the following formula:

$$\%\;\mathrm{Inhibition}\;=\frac{\left(\mathrm{OD}\;\mathrm{control}-\mathrm{OD}\;\mathrm{sample}\right)}{\mathrm{OD}\;\mathrm{control}}\;\;\times \;100$$

Trapping percentages and the CI_50_ (free radical scavenger concentration required to neutralize 50% of free radicals) were calculated using GraphPad Prism software 8.0.1 (244).

#### 2.4.1 Total Antioxidant Capacity Investigation

The total antioxidant capacity of the aqueous *P. africanum* extract was determined using the method of Prieto et al. (1999). The antioxidant capacity of the *P. africanum* extract was determined by mixing 0.3 mL with a reagent solution and incubating the mixture at 95°C for 90 min. The antioxidant capacity was expressed in milligram of ascorbic acid equivalents per gram of dry matter (mg EAA/g DW).

#### 2.4.2 DPPH Assay

The DPPH test is a colorimetric method used to detect the reduction of the DPPH radical, indicated by a loss of color between 470 and 517 nm. The protocol used for DPPH radical trapping is based on Bassene's method (2012). *P. africanum* extract was diluted for the experiment to obtain final concentrations of 500, 250, 125, 62.5, 31.25, 15.625, and 7.8125 µg/mL. A total of 25 µL of each dilution was added to the wells, followed by 75 µL of DPPH (0.02%) solution. After a 30‐min incubation in the dark at room temperature, optical densities were measured at 517 nm. The negative control comprised DPPH without extract, while the positive control involved ascorbic acid treated under the same conditions with final concentrations of 50, 25, 12.5, 6.25, 3.125, 1.5625, and 0.78125 µg/mL. Additional tests were performed with the extracts under the same conditions to observe fluorescence. All tests were performed in triplicate.

#### 2.4.3 ABTS Assay

We used the ABTS+ cation radical decolorization test described by Khan et al. (2012) to evaluate anti‐radical activity. This involved reacting ABTS with potassium permanganate (KMnO_4_) or potassium persulfate (K_2_S_2_O_8_) to form the ABTS+ radical, which changes color from blue to green. The antioxidant capacity was measured by comparing test compounds to ascorbic acid. *P. africanum* extract was diluted to final concentrations of 500, 250, 125, 62.5, 31.25, 15.625, and 7.8125 µg/mL. Optical densities were read at 734 nm after incubation, and tests were carried out in triplicate. The negative control used ABTS reagent without extract, and the positive control involved different ascorbic acid concentrations.

#### 2.4.4 FRAP Assay

The Fe^3+^ reduction test was carried out according to the protocol described by Path (1994). It is used to measure the ability of a substance to reduce Fe^2+^ ions and form a red‐orange complex with measurable optical density at 505 nm. In a 96‐well microplate, 196 µL of distilled water was added to the first column and 100 µL to each subsequent column for dilution. Then, 4 µL of each prepared extract at 100 mg/mL was added and diluted with a dilution factor of 2 to reach a final volume of 100 µL. The test involved mixing 25 µL of extract and 25 µL of FeSO_4_ solution, followed by a 15‐min incubation at room temperature away from light. After this, 50 µL of the 1,10‐phenanthroline solution was added, and the plates were re‐incubated for 15 min, again at room temperature. The optical density of the wells' contents was then measured at 505 nm using a plate reader (TECAN M200). The negative control consisted of 25 µL of methanol + 25 µL of FeSO_4_ solution + 50 µL of 1,10‐phenanthroline.

### 2.5 Animal Material and Ethical Statement

The animals used in this study were pups and adult albino Wistar rats, both male and female. The adult rats had an average weight of 180 g and were aged 8–10 weeks. These rats were bred in the animal facilities at the University of Yaoundé I. They were housed in cages lined with wood shavings, maintained at ambient temperature with sufficient ventilation, and subjected to a natural light‐dark cycle. The rats had free access to food and tap water throughout the experiment. The pups were raised under identical conditions. All experiments conducted in this study adhered to the guidelines of the European Union on Animal Care (CEE Council 86/609), which were endorsed by the Cameroon Institutional National Ethics Committee under the Ministry of Scientific Research and Technology Innovation (Reg. number FWA‐IRD 0001954).

### 2.6 Experimental Model of Autism

#### 2.6.1 Preparation of Sodium Valproate and Induction of ASD

To induce ASD in rat, the following procedure was followed. After confirmation of gestation, the rats were each isolated in their cage. On post‐gestation days 11, 12, and 13, 27 female rats received a single oral dose by gavage of 800 mg/kg sodium valproate, and six females received a dose of 10 mL/kg distilled water. At the end of this treatment, six miscarriages (fetal resorptions), chromodacryorrhea (a red crust of dry tears due to stress‐induced overproduction of porphyrin), and two late‐gestation deaths were observed in the female treated with sodium valproate (VPA). Thus, there were 25 pregnant rats, of which 19 were treated with VPA and 6 with distilled water.

#### 2.6.2 Selection Criteria and Allocation of Offspring Rats

After birth (21st day post‐natal), young males were separated from females, weaned, and subjected to a series of behavioral tests for 6 days. Tests of sociability, stereotypy, anxiety, and pain sensitivity assessed behavioral disorders. Inclusion criteria for each test were scored concerning the mean responses of normal animals receiving distilled water. Thus, for the 3‐chamber social interaction test, animals whose interaction time with the old and new conspecific was less than 10 and 20 s, respectively, were considered to have developed autism. For the stereotypy test, rats were considered to present autistic disorders if the number of groomings and the number of burials exceeded 20 and 5, respectively. Considering these factors, 20 ASD rats were selected from the 37 offspring male rats of VPA‐treated females. At the end of this series of selection tests, the ASD rats were weighed, marked, and divided into four batches of five animals each. The 10 offspring male rats from the untreated female rats were labeled neurotypical animals and divided into two groups treated respectively with distilled water (NTC) or the plant extract at 190 mg/kg (NT‐190). The number of animals per group was determined by using the formula of the degree of freedom of analysis of variance “E.” It is equal to the total number of animals minus the number of groups according to Charan and Kantharia (2013). ASD animals were treated for 15 days: distilled water (ASD), bumetanide at 4 mg/kg (ASD‐B), and *P. africanum* 190 and 760 mg/kg (ASD‐190 and ASD‐760).

### 2.7 Assessment of Autistic Behavior

After each animal had passed through a device, it was cleaned with 70% alcohol.

#### 2.7.1 Three‐Chamber Social Paradigm Test 3‐CSP

It was conducted according to Kaidanovich‐Beilin et al. (2011) based on the free choice of the animals to move from one compartment to another to evaluate their interaction, social preference, and memory. The test was conducted in three phases. **Phase 1**: The test animal was placed in the central chamber for 1 min. Access to the other two chambers was free to allow the animal to explore and become accustomed to the environment. The parameter evaluated was the time taken to leave the central compartment. **Phase 2**: The test animal was confined in the central compartment, and an unfamiliar conspecific of the same sex and age was placed in the restraint box in the left‐hand compartment. The doors were opened for 5 min to allow the test animal to explore the other two chambers. The sociability index expressed sociability as the ratio of the time spent interacting with the conspecific to the time spent in the empty compartment. **Phase 3**: With the test animal and the former conspecific locked in their respective compartments, a new, unfamiliar conspecific of the same sex and age was placed in the empty correct compartment restraint box. The doors were reopened for 5 min. Preference for social novelty was expressed by the social novelty preference index, which is the ratio of time spent interacting with the new conspecific to time spent interacting with the old conspecific.

#### 2.7.2 Stereotypy Test

This test evaluates the autistic character marked by repetitive animal movements. It was performed following the procedure laid out by Oksana et al. (2011). To study stereotypy, each animal was placed in the device. They were observed and filmed for 5 min. The number of groomings and burials were evaluated.

#### 2.7.3 Elevated plus Maze Test

According to the procedure described by Degroote (2016), animal aversion to open spaces to evaluate the level of anxiety has been highlighted in this test. Each animal was placed in the center of the device, and its attitude and movements were observed for 5 min. The parameters evaluated were as: time spent in the open arms in seconds; number of entries into the open arms; time spent in the closed arms in seconds; number of entries into the closed arms; number of “sit‐ups” when the animal stands up on its hind legs and leans on the edges of the device; number of “head drops” when the animal bends over and puts its head over the edge of an open arm; and number of “groomings” when the animal cleans its body.

#### 2.7.4 Open Arena Test

This test assessed the motor skills and the emotional reactivity of animals toward a new and spacious environment to highlight some psychotropic actions. The Taiwe et al.'s (2015) procedure was used to conduct this test. The animal was placed in the center of a well‐lit device, allowing it to explore freely for 5 min. The parameters evaluated were: time spent in the center of the device in seconds; number of lines crossed; number of “sit‐ups” or number of times the animal stood up on its hind legs and leaned on the edges of the device; and number of “groomings” or number of times the animal cleaned its body.

#### 2.7.5 Hot Plate Test

This test, as directed by Eddy and Leimback (1953), was made to measure the reactivity of animals to heat‐induced pain. The animal was placed on a plate previously heated to 55 ± 5°C, and the withdrawal and licking of the animal's paw was observed after a few seconds.

### 2.8 Sacrifice and Sample Preparation

After the last test, all animals were sacrificed by decapitation after ether anesthesia. Brains were removed with fine scissors and forceps, washed in 0.9% NaCl, wrung out on absorbent paper, and weighed on a balance (Mettler PL 301). For each batch, three brains were used for homogenates, and two brains were stored in 10% buffered formalin for subsequent histological sections. The hippocampus, cerebellum, amygdala, and prefrontal cortex were harvested, weighed, and ground separately to prepare the homogenates. For this purpose, each ground organ was introduced into dry, labeled tubes, to which 2 mL of Tris buffer was added. The mixture was centrifuged at 3000 rpm for 25 min. The supernatant was pipetted into a labeled Eppendorf tube and stored at −20°C for subsequent biochemical assays.

### 2.9 Estimation of some Biochemical Markers

The amount of gamma‐aminobutyric acid (GABA) in the homogenate was assessed using the colorimetric assay technique described by Lowe et al. (1958), and the activity of L‐glutamate decarboxylase (L‐GAD) and GABA‐transaminase (GABA‐T) was assessed using the colorimetric assay method of Nayak and Chatterjee (2001). Serotonin levels in homogenate were estimated using the method described by Schlumpf et al. (1974). Superoxide dismutase (SOD) activity was assessed using the method of Misra and Fridovish (1972). Total reduced glutathione (GSH) and malondialdehyde (MDA) content were evaluated using the colorimetric assay techniques described by Ellman (1959) and Wilbur et al. (1949), respectively.

### 2.10 Histological Analysis and Neuron Counting

Histological analysis involves the preparation of tissues for microscopic observation. After 2 weeks of fixation in 10% buffered formaldehyde, the organs (hippocampus, amygdala, and cerebellum) were trimmed and dehydrated. Subsequently, they were immersed in xylene and embedded in molten paraffin solution at 60°C for 5 h. Serial sections of 5 µm were then obtained from the paraffin blocks containing the tissues using a Reichert‐Jung 2030 microtome and stained with hematoxylin and eosin (H&E). The stained sections were examined, and images were captured using a Leitz Wetzlar Germany 513 light microscope connected to a Celestron 44.421 digital camera linked to a computer. Image J software (version 1.4.3.67) was utilized to perform histomorphometric evaluations (neuron counting) in the dentate gyrus, CA1, and CA3 regions of the hippocampus and the amygdala. Neurons always have visible cytoplasm around the nucleus. Neuron counting involves identifying neuron nuclei, typically large and round and characterized by a distinct nucleolus. For neuron counting, images of the left hemisphere of each animal were captured, and neurons were identified and counted using Image J. To ensure accuracy, identical sections from the same areas were carefully and randomly selected for our sample photographs across all groups. Several key characteristics were noted to identify the hippocampus and amygdala in a rat histological section. First, the medial temporal lobe was located near the lateral ventricle. Next, the hippocampus was identified as a curved and densely packed structure with distinct layers, including CA1, CA3, and the dentate gyrus. The amygdala, situated within the temporal lobe and adjacent to the hippocampus, is recognized by its closely packed neurons and typically appears as a smaller, rounded structure compared to the hippocampus (Kjonigsen et al. 2015; García‐Cabezas et al. 2016).

### 2.11 Statistical Analyses

Statistical analyses of the values obtained were carried out using GraphPad Prism 8.0.1 software. Results were expressed as mean ± standard error on the mean (SEM) or mean ± standard deviation (SD), and the different values were compared using the “one‐way ANOVA” analysis of variance test, followed by Tukey's multiple comparison posttest. Differences were considered significant at *p* < 0.05.

## 3 Results

### 3.1 Survival and Identification of ASD Model

**FIGURE 1:**
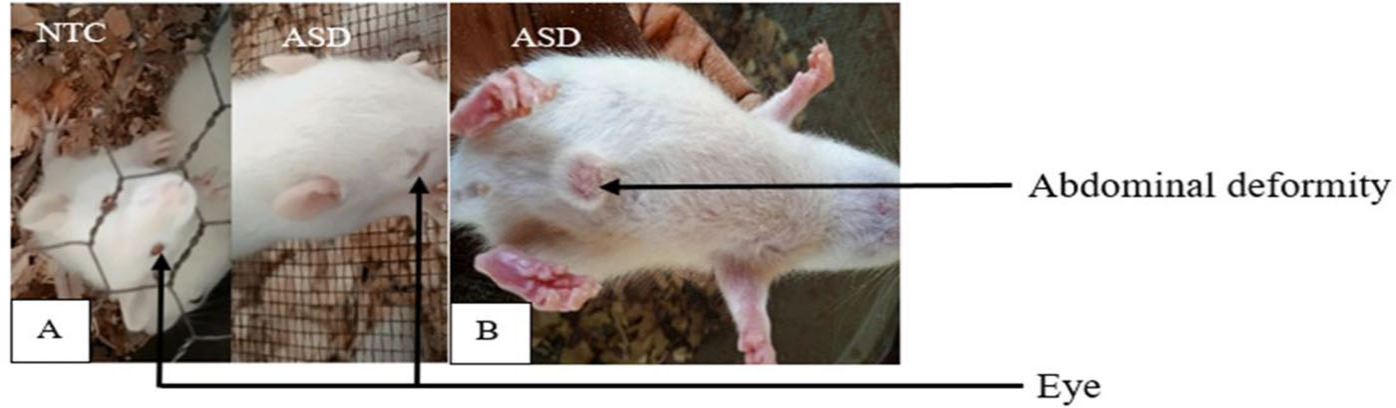
Some malformations: (A) delayed eye‐opening and (B) abdominal deformity. NTC: neurotypical animals treated with distilled water (10 mL/kg). ASD: ASD animals treated with distilled water (10 mL/kg).

Malformations were observed in the offspring after the administration of 800 mg/kg valproate to females during days 11, 12, and 13 of gestation. Out of 37 rats, 10 showed a 7‐day delay (21 days postnatal) in eye‐opening, compared to normal rats that opened their eyes 14 days after birth. Additionally, one animal displayed an abdominal deformity (Figure 1).

### 3.2 Quantitative Phytochemical Assays

To assess the phytochemical composition of *P. africanum* aqueous extract, the concentrations of secondary metabolites that can specify the plant profile were determined. The water extract of the bark sapwood of *P. africanum* was analyzed, and bioactive compounds were revealed (Table 1). The results showed that *P. africanum* has appreciable concentrations of flavonoids (345.68 ± 3.06 mg QE/g), polyphenols (456.09 ± 2.45 mg GAE/g), and tannins (54.02 ± 0.08 mg TAE/g).

**TABLE 1:** Quantitative phytochemical screening of aqueous extract of *Piptadeniastrum africanum*.

Secondary metabolites	Aqueous extract of the bark sapwood of *P. africanum*
Flavonoids (mg QE/g)	345.68 ± 3.06
Polyphenols (mg GAE/g)	456.09 ± 2.45
Tannins (mg TAE/g)	54.02 ± 0.08

*Note*: Values expressed are means ± SD. *n* = 3.

Abbreviations: GAE, gallic acid equivalent; TAE, tannic acid equivalent; QE, Quillaja equivalent.

### 3.3 UHPLC‐MS Outcomes

**FIGURE 2:**
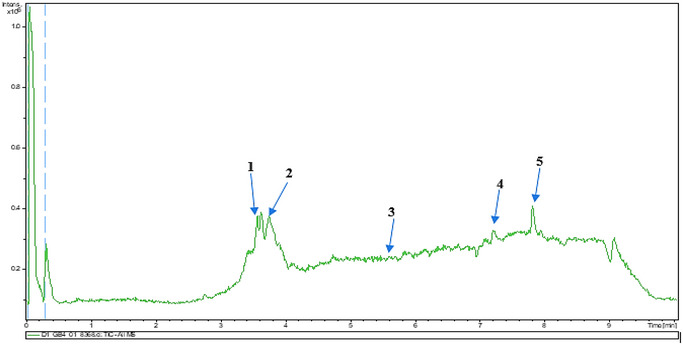
Identification spectrum of aqueous extract of *Piptadeniastrum africanum*.

The UHPLC‐MS analysis of the aqueous extract from *P. africanum* revealed the presence of specific molecules. The identification spectrum is illustrated in Figure 2, while the mass spectrometry data and structures are provided in Table 2. One of the identified molecules is dimethoxy‐trihydroxy(iso)flavone isomer 1,3,3′‐di‐O‐methyl ellagic acid, methoxy‐tetrahydroxy(iso)flavone, β‐Sitostenone, 5α‐Stigmast‐7,22‐dien‐3‐one.

**TABLE 2:** UHPLC‐MS identification of five active molecules in the aqueous extract of *Piptadeniastrum africanum*.

Compound name	Molecular ion	Mass spectrum and structures
1‐Dimethoxy‐trihydroxy(iso)flavone isomer 1 (m/z = 330.29)	[M−H] Theoretical values = 329.28 Experimental values = 329.30	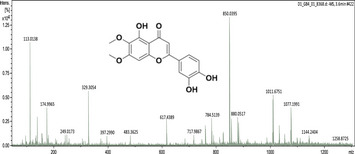
2‐3,3′ ‐Di‐O‐methyl ellagic acid (m/z = 330.24)	[M−H] Theoretical values = 329.23 Experimental values = 329.30	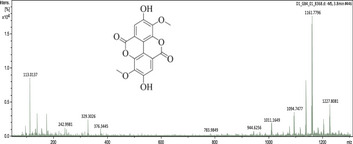
3‐ Methoxy‐tetrahydroxy(iso)flavone (m/z = 316.26)	[M−H] Theoretical values = 315.25 Experimental values = 315.32	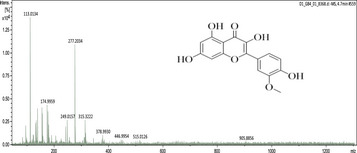
4‐β‐Sitostenone (m/z = 412.7)	[M+Na−2H] Theoretical values = 433.67 Experimental values = 433.35	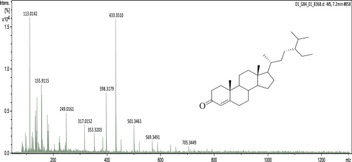
5‐5α‐Stigmast‐7,22‐dien‐3‐one (m/z = 410.7)	[M−H] Theoretical values = 409.69 Experimental values = 409.39	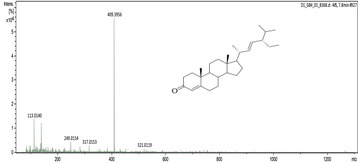

Abbreviation: m/z, mass to charge ratio.

### 3.4 Molecular Docking of H3R (7F61) and HDAC2 (5ix0)

Table 3 presents the molecular docking scores of compounds identified with H3R (7F61) and HDAC2 (5ix0). Concerning the H3R protein, the highest MOE score function was applied to the tested compounds to evaluate their binding affinities (Table 4). The compounds showed good binding affinity values ranging from −4.97 kcal/mol (3,3′‐di‐O‐methyl ellagic acid) to −5.93 (dimethoxy‐trihydroxy (iso) flavone isomer 1). However, the score of our ligands was higher than that of the reference compounds (ciproxifan) except for 3,3′‐di‐O‐methylellagic acid. The considerable RMSD value of <2 was attributed to the high stability of these compounds in the binding site (Table 3). Table 5 shows the different interactions of the compounds of interest with H3R (7F61) and HDAC2 (5ix0). For the H3R protein, the compounds interacted with important residues in the active pocket of the target. However, most ligands interacted with isomer 1 of dimethoxy‐trihydroxy(iso)flavone (His 187, Glu 395), 3,3′‐di‐O‐methylllagic acid (His 187), methoxy‐tetrahydroxy(iso)flavone (Glu88, Ser 182, Tyr194), β‐Sitostenone (Lys 95), and 5α Stigmast‐7,22‐dien‐3‐one (Lys 95) from the active pocket of the target. In addition to these interactions, interactions with residues Asn99, Arg98, Tyr194, and Asn11 similar to those obtained with ciprofaxin were also recorded with dimethoxy‐trihydroxy(iso)flavone. About the HDAC2 protein, the table presents the molecular docking scores of compounds identified with HDAC2 (5ix0). The compounds showed good binding affinity values ranging from −3.2 kcal/mol (β‐Sitostenone) to −6.9 (Dimethoxy‐trihydroxy (iso) flavone isomer 1). However, the dimethoxy‐trihydroxy (iso) flavone isomer 1 score exceeded the reference compounds. The considerable RMSD value of <2 was attributed to the high stability of these compounds in the binding site (Table 3). About the HDAC2 protein, Table 5 shows the different interactions of the compounds of interest and HDAC2. The compounds dimethoxy‐trihydroxy(iso)flavone isomer (Asp269), 3,3′‐di‐O‐methylellagic acid (Leu 276), Methoxy‐tetrahydroxy(iso)flavone (Asp 181, Asp 269), and β‐Sitostenone exhibited hydrophobic, hydrogen, pi‐cation, and pi‐pi interactions with the important residues of the active pocket of the enzyme. In addition, compared to tantacruzamate, dimethoxy‐trihydroxy(iso)flavone isomer 1 exhibited numerous hydrophobic, pi‐pi, and hydrogen interactions with residues Phe210, His146, Cys156, Gly154, Met35, Tyr128, His183, and Phe15 similar to that of the reference.

**TABLE 3:** Simulated molecular docking energies of the tested ligands with H3R and HDAC2.

Parameters	Docking energies (Kcal/mol)		RMSD	
Compounds names	H3R	HDAC2	H3R	HDAC2
Dimethoxy‐trihydroxy(iso)flavone isomer 1	−5.93	−6.90	1.06	1.22
3,3′‐Di‐O‐methyl ellagic acid	−4.97	−4.98	1.31	1.25
Methoxy‐tetrahydroxy(iso)flavone	−5.54	−4.79	2.96	1.96
β‐Sitostenone	−5.57	−3.20	1.93	1.35
5α‐Stigmast‐7,22‐dien‐3‐one	−5.81	−4.79	2.01	2.04
Ciproxifan	−5.15	/	1.03	/
Santacruzamate	/	−6.71	/	1.05

**TABLE 4:** 2D and 3D views of the interaction profiles of compounds and H3R in the MOE viewer.

3D	2D	Name compounds
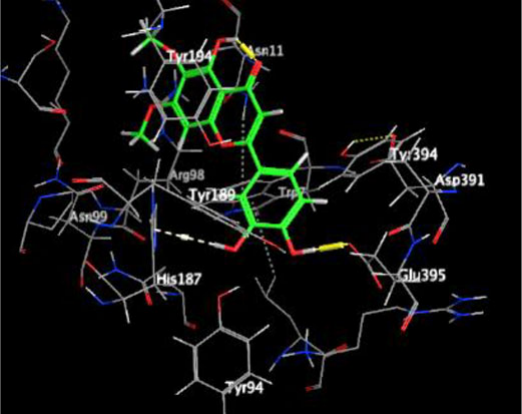	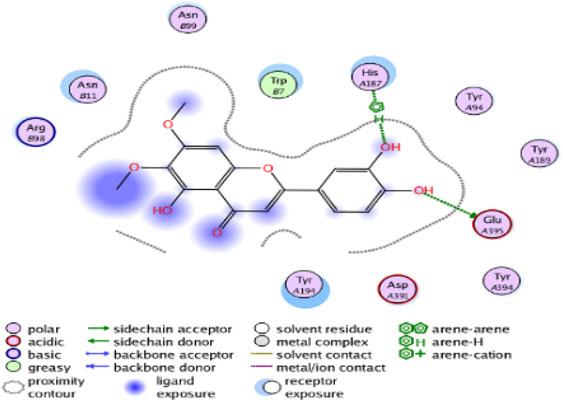	Dimethoxy‐trihydroxy(iso)flavone isomer 1
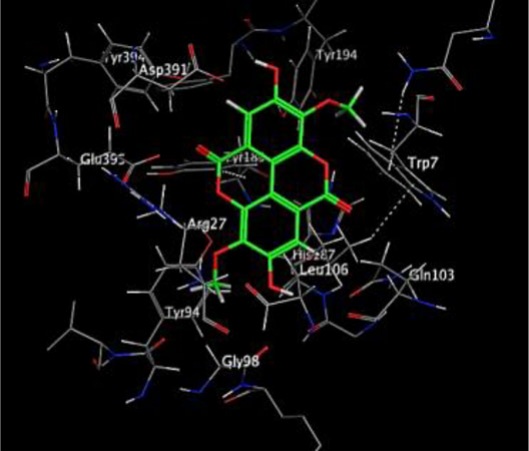	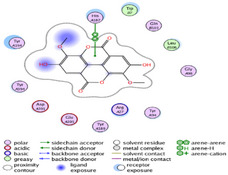	3,3′‐Di‐O‐methyl ellagic acid
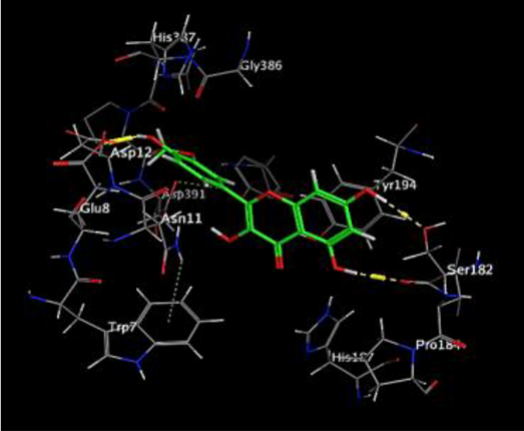	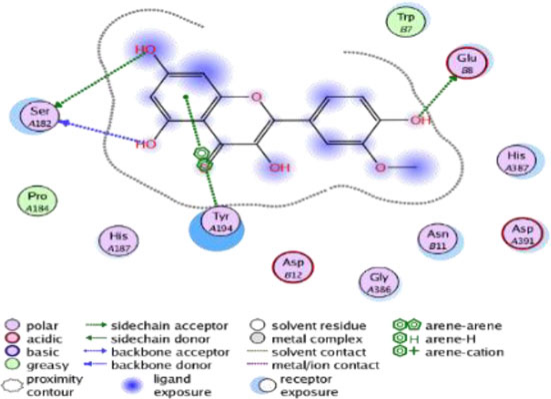	Methoxy‐tetrahydroxy(iso)flavone
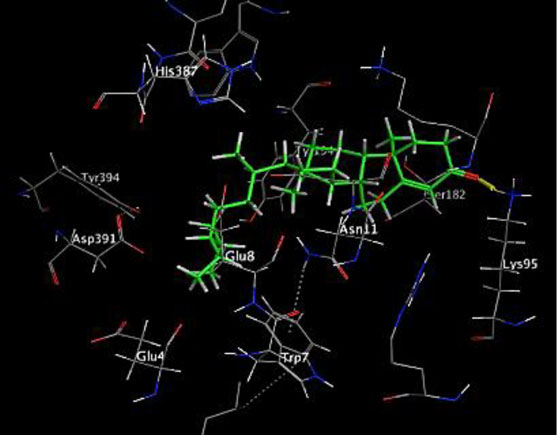	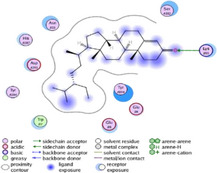	β‐Sitostenone
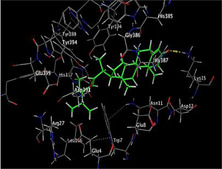	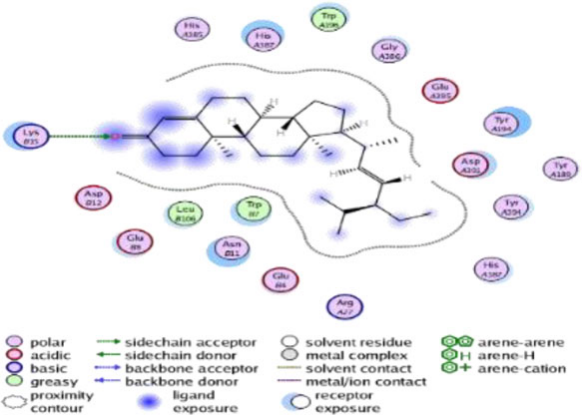	5α‐Stigmast‐7,22‐dien‐3‐one
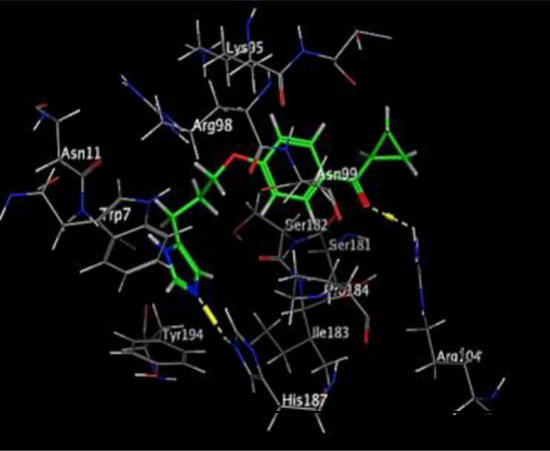	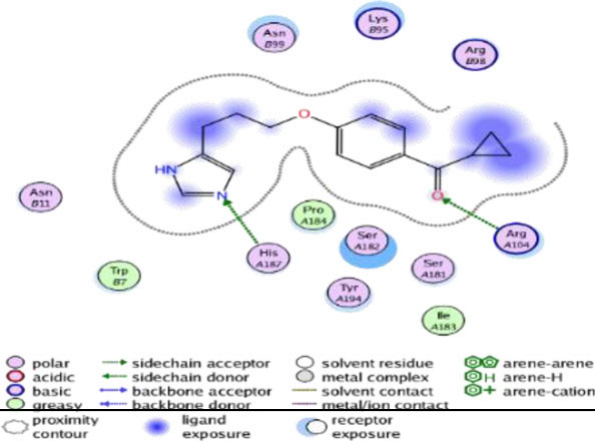	Ciproxifan

**TABLE 5:** 2D and 3D views of the interaction profiles of compounds and HDAC2 in the MOE viewer.

3D	2D	Names compounds
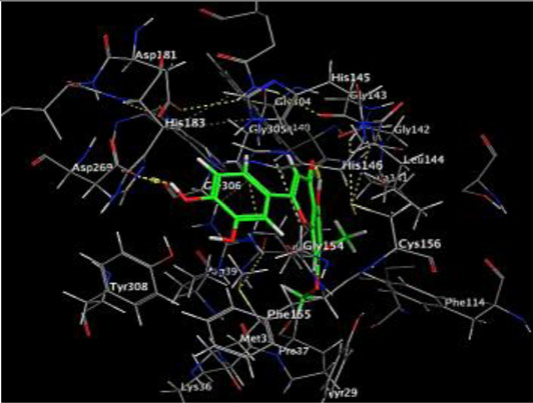	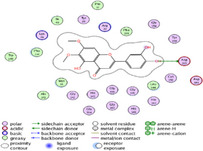	Dimethoxy‐trihydroxy(iso)flavone isomer 1
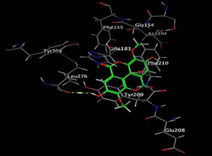	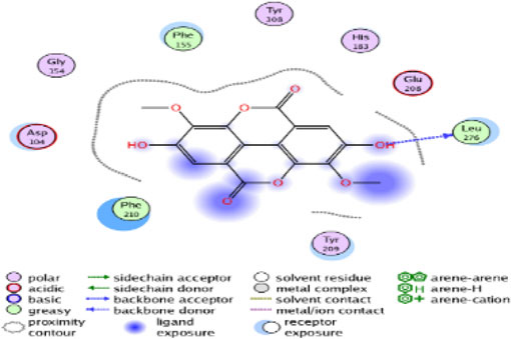	3,3′‐Di‐O‐methyl ellagic acid
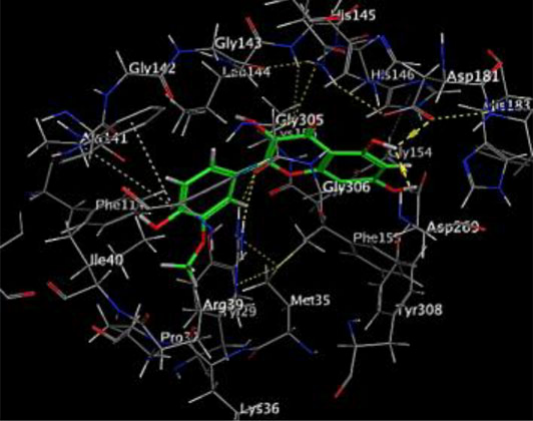	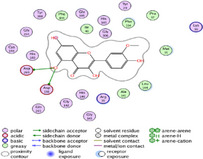	Methoxy‐tetrahydroxy(iso)flavone
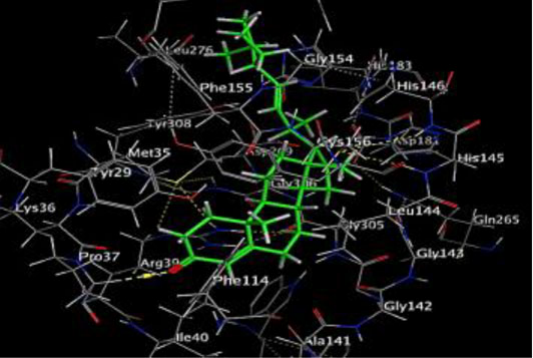	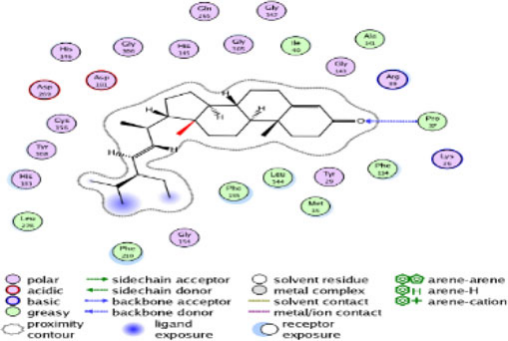	β‐Sitostenone
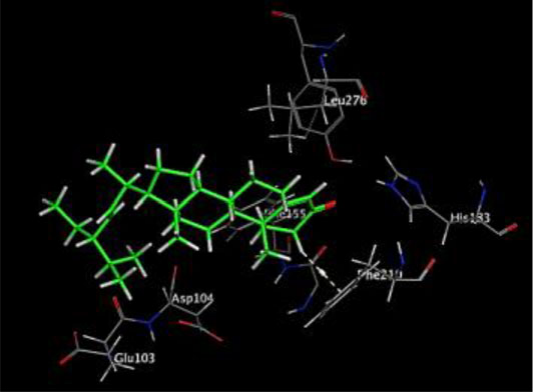	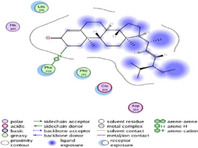	5α‐Stigmast‐7,22‐dien‐3‐one
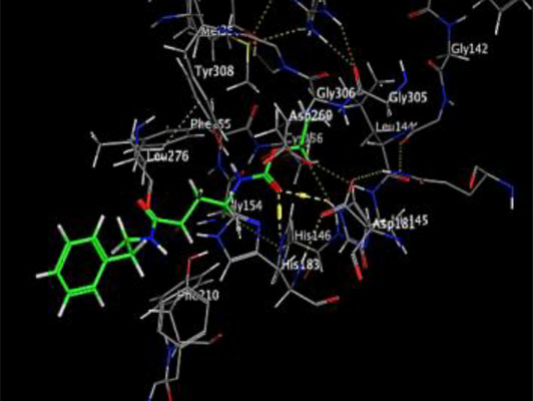	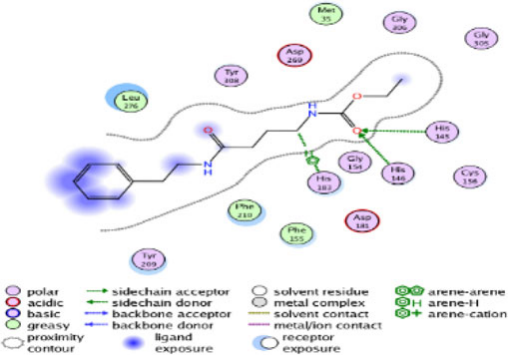	Tantacruzamate

### 3.5 In Vitro Antioxidant Potential

The antioxidant activity of the aqueous *P. africanum* extract was assessed, and the results are presented in Table 6. The data indicate that the total antioxidant capacity depends on the concentration of the *P. africanum* extract, with a maximum value of 10.38 ± 0.29 µg EE of ascorbic acid/g ms at 1000 µg/mL. Furthermore, there was a significant inhibitory activity concerning the concentration of the DPPH and ABTS, reaching a maximum value of 75.79 ± 1.19% at a concentration of 1000 µg/mL with an IC_50_ of 98.78 µg/mL for DPPH and 72.20 ± 1.61% at a concentration of 1000 µg/mL with an IC_50_ of 499.65 µg/mL for ABTS. Similarly, vitamin C demonstrated concentration‐dependent inhibition of both DPPH and ABTS, with values of 98.79 ± 1.16% at 1000 µg/mL and an IC_50_ of 120.66 µg/mL for DPPH and 99.84 ± 1.31% at 1000 µg/mL with an IC_50_ of 191.19 µg/mL for ABTS. Table 6 displays the concentrations of the antioxidant activity of the aqueous extract from the bark of *P. africanum* against the FRAP radical. The results demonstrate a significant inhibitory activity based on the concentration of the FRAP activity, with the inhibition reaching a maximum value of 13.02 ± 0.17% at a concentration of 1000 µg/mL.

**TABLE 6:** Scavenging effects of *P. africanum* extract on DPPH, ABTS, and FRAP radicals.

Concentration (µg/mL)		1000	800	600	400	200	100	IC_50_ (µg/mL)
TAC (µg EE of ascorbic acid/g MS)		10.38 ± 0.29	8.31 ± 0.35	6.24 ± 0.46	4.13 ± 0.40	2.39 ± 0.48	1.39 ± 0.46	/
DPPH (%)	*P. africanum*	75.79 ± 1.19	58.07 ± 0.22	56.90 ± 0.22	55.35 ± 0.36	54.79 ± 0.07	52.99 ± 0.30	98.78
	Vit C	98.79 ± 1.16	88.07 ± 0.10	86.90 ± 0.22	78.38 ± 0.36	64.79 ± 0.08	42.99 ± 0.30	120.66
ABTS (%)	*P. africanum*	72.20 ± 1.61	55.58 ± 2.28	47.45 ± 1.01	40.80 ± 1.52	36.66 ± 3.50	21.86 ± 0.84	499.65
	Vit C	99.84 ± 1.31	88.58 ± 2.23	77.35 ± 1.01	64.84 ± 1.52	56.66 ± 3.50	31.66 ± 0.33	191.19
FRAP (mEAG/g)	*P. africanum*	13.02±0.17	6.87± 0.32	6.08± 0.07	5.28 ± 0.10	4.46± 0.08	3.01± 0.03	/

Abbreviations: ABTS, 2,2′‐azino‐bis‐(3‐ethylbenzothiazoline‐6‐sulfonic) acid); DPPH, 2,2‐diphenyl‐1‐picrylhydrazyl; FRAP, ferric reducing antioxidant power; IC_50_, inhibitory concentration 50; ′mgEAG, milligram equivalent of gallic acid.

### 3.6 *P. africanum* Effects on Some Behavioral Disorders

#### 3.6.1 Effects on Exploratory Activity and Social Interaction

**FIGURE 3:**
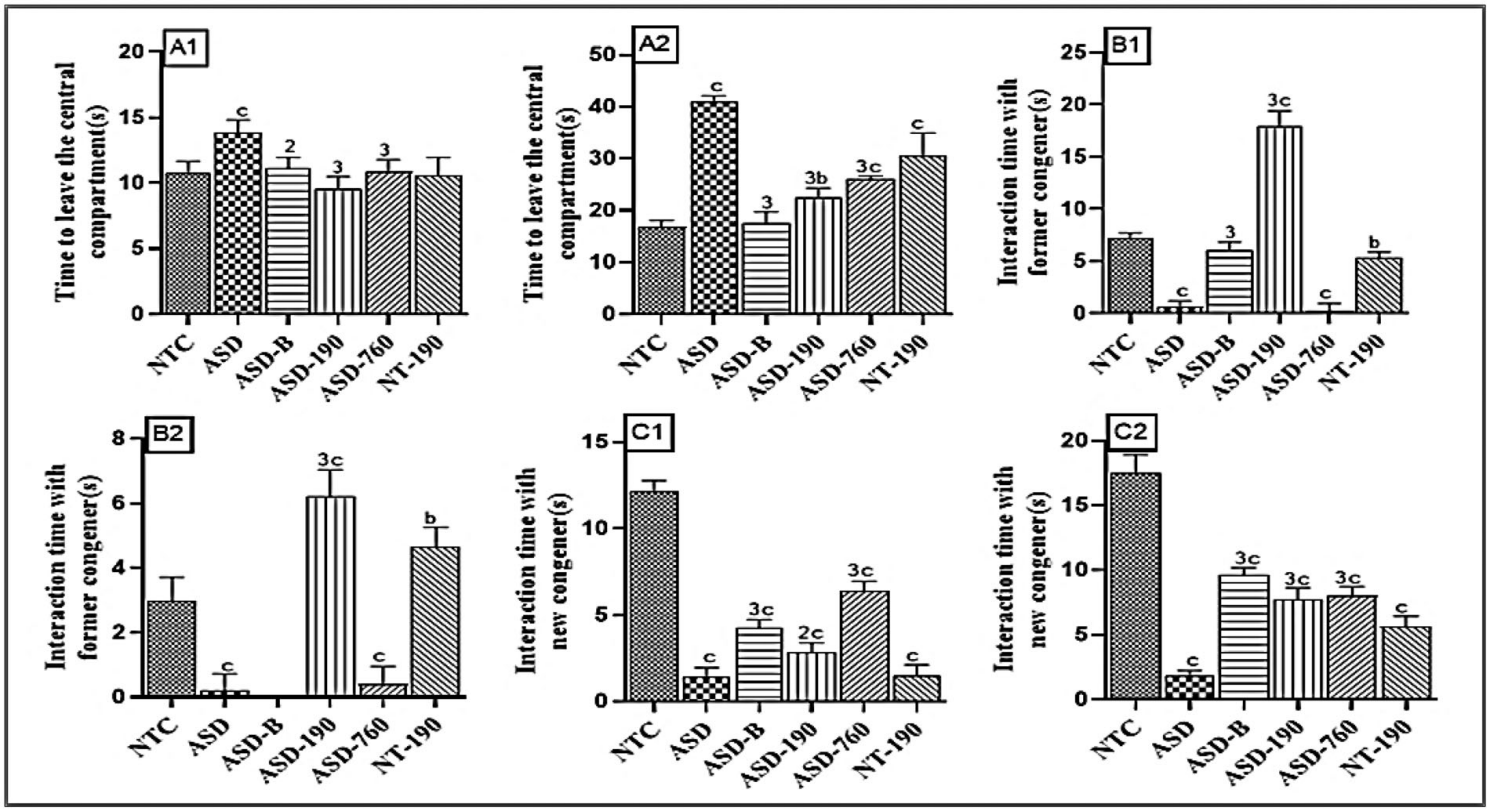
Effects of *Piptadeniastrum africanum* on exploratory activity (A1, A2) and social interaction (B1, B2 and C1, C2). Each bar represents the mean ± MSE; *n* = 5. Each bar represents the mean ± MSE; *n* = 5. ^a^ *p <* 0.05; ^b^ *p <* 0.01; ^c^ *p <* 0.001: significant differences versus NTC. ^1^ *p <* 0.05; ^2^ *p <* 0.01; ^3^ *p <* 0.001: significant differences versus ASD. NTC: neurotypical animals treated with distilled water (10 mL/kg); ASD: ASD animal treated with distilled water (10 mL/kg); ASD‐B: ASD animal treated at Bumetanide (4 mg/kg); ASD‐190, ASD‐760: ASD animals treated with the aqueous extract *P. africanum* at doses of 190 and 760 mg/kg; NT‐190: neurotypical animal treated with the aqueous extract from *P. africanum* at dose of 190 mg/kg. 1 and 2 designate waves 1 and 2, respectively.

The data depicted in Figure 3 is derived from three‐chamber social paradigm tests. In Figure 3A, we can observe the effects of *P. africanum* on the duration it takes for the subjects to exit the central compartment of the sociability cage. Fetuses exposed to sodium valproate (VPA) on gestation days 11–13 need more time to leave the central compartment, with an increase of 28.5% (*p* < 0.001; mean: 13.88; SD: 0.93) after 28 days (wave 1) and 59.09% (*p* < 0.001; Mean: 41.07; SD: 1.09) after 37 days (wave 2). Notably, *P. africanum* extract led to a reduction in the time taken to exit the central compartment for subjects exposed to VPA, indicating an increase in exploration activity. It also extended the interaction time with the former congener and rectified the reduced preference for social novelty. Additionally, administering *P. africanum* extract at 190 mg/kg amplified this interaction by 18‐fold (*p* < 0.001; Mean: 17.96; SD: 1.42). Furthermore, Bumetanide enhanced interaction by 89.64% (*p* < 0.001; Mean: 6.10; SD: 0.76). Meanwhile, the negative control group exhibited a decreased preference for social novelty in both waves.

#### 3.6.2 Effects on Stereotyped Behavior

**FIGURE 4:**
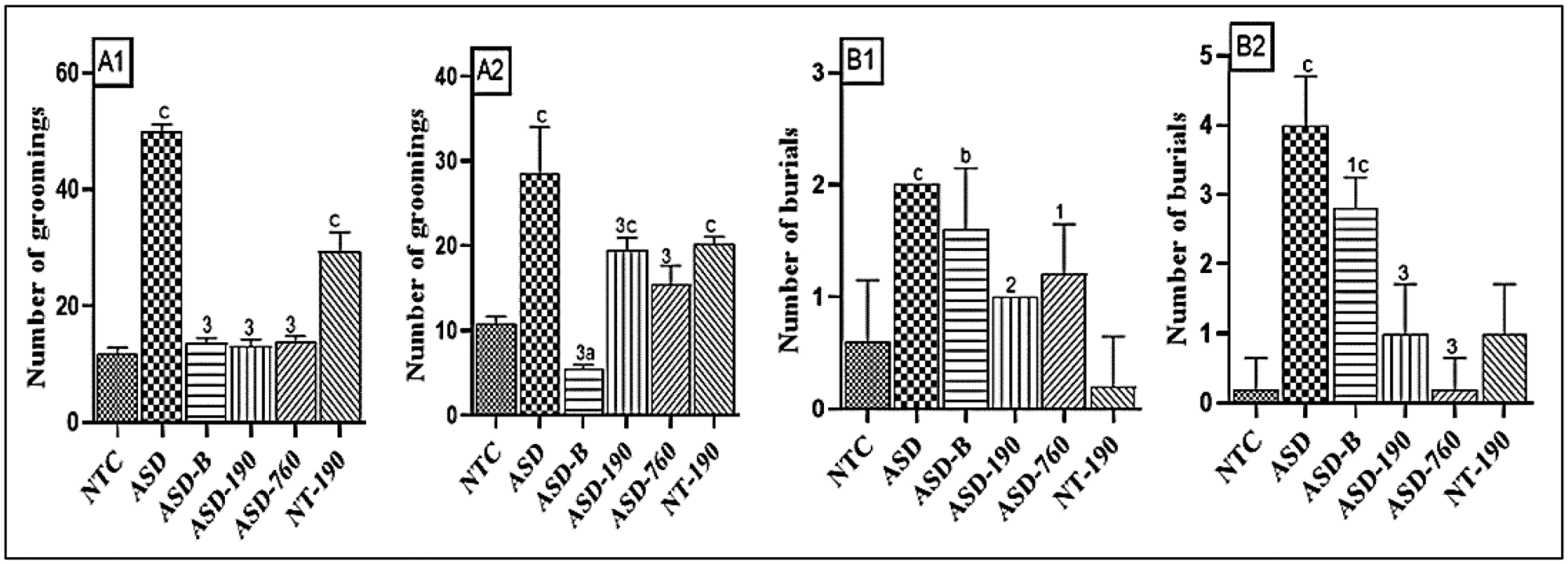
Effects of *Piptadeniastrum africanum* aqueous extract on groomings (A1, A2) and burials (B1, B2). Each bar represents the mean ± MSE; *n* = 5. ^a^ *p <* 0.05; ^b^ *p <* 0.01; ^c^ *p <* 0.001: significant differences versus NTC. ^1^ *p <* 0.05; ^2^ *p <* 0.01; ^3^ *p <* 0.001: significant differences versus ASD. NTC: neurotypical animals treated with distilled water (10 mL/kg); ASD: ASD animal treated with distilled water (10 mL/kg); ASD‐B: ASD animal treated at bumetanide (4 mg/kg); ASD‐190, ASD‐760: ASD animals treated with the aqueous extract *P. africanum* at doses of 190 and 760 mg/kg; NT‐190: neurotypical animal treated with the aqueous extract from *P. africanum* at the dose of 190 mg/kg. 1 and 2 designate waves 1 and 2, respectively.

Figure 4A,B demonstrate a notable increase in grooming and burials 28 days (wave 1) and 37 days (wave 2) after weaning. The negative control for wave 1, with a Mean of 50.00 and SD of 1.22, exhibited a 70%–76.4% increase in grooming and a 62.23% to 4‐fold increase in burrowing (Mean: 4.00; SD: 0.70) compared to the NTC in wave 2. The *P. africanum* extract at 190 and 760 mg/kg, as well as bumetanide, reduced grooming by 72.4%–73.6% in wave 1 and 32.16%–81.11% in wave 2 compared to the negative control. Moreover, the 190 (Mean: 1; SD: 0) and 760 mg/kg doses of the plant extract in wave 1 resulted in a 40%–50% decrease in burials compared to the ASD control. In wave 2, the 190 and 760 (Mean: 0.20; SD: 0.44) mg/kg doses, along with bumetanide, showed a 30%–75% decline compared to the ASD control. Additionally, the pharmacological control demonstrated an increase in the number of groomings in both waves compared to the NTC control.

#### 3.6.3 Effects on Exploratory Activity and Anxiety in the Open Arena Test

The open arena test (OA) is a commonly used ethological approach to evaluate anxiety‐like behavior in murine model exposure to sodium valproate in utero. Data of OA are presented in Table 7. For wave 1, the time spent in the center of the arena by rats in the ASD group (2.20 ± 0.20 s) was significantly reduced compared to NTS animals (1.19 ± 0.02 s). However, *P. africanum* extract with a 190 mg/kg dosage increased the time spent by 40.51% for wave 1 and 68.75% for wave 2. The negative control group showed a significant increase in turnarounds compared to NTC. In the present study, the aqueous extract of *P. africanum* at 190 and 760 mg/kg reduced the number of turnarounds. Bumetanide also reduced the number of turnarounds by 30% (*p* < 0.001) in wave 1 and by 40% (*p* < 0.05) in wave 2 compared to ASD animals.

**TABLE 7:** Effects of *Piptadeniastrum africanum* aqueous extract on exploratory activity and anxiety.

Groups	Time spent at the center (s)	Number of regressions	Number of lines crossed	Number of groomings
NTC	2.20 ± 0.20 (1.07 ± 0.07)	4.00 ± 0.31 (3.60 ± 0.24)	36.60 ± 1.12 (50.20 ± 1.77)	11.80 ± 0.48 (13.20 ± 0.20)
ASD	1.19 ± 0.02^2^ (1.00 ± 0.00)	8.00 ± 0.31^c^ (6.40 ± 0.24)^c^	23.40 ± 0.60^c^ (20.60 ± 0.60)^c^	27.40 ± 2.20^c^ (28.00 ± 1.89)^c^
ASD‐B	1.60 ± 0.24 (0.95 ± 0.16)	5.60 ± 0.24^3a^ (5.80 ± 0.20)^c^	50.60 ± 0.60^3c^ (30.80 ± 2.05)^2c^	18.60 ± 0.92^3b^ (21.60 ± 1.03)^1b^
ASD‐190	2.00 ± 0.01 (3.20 ± 0.12)^3c^	4.60 ± 0.24^3^ (5.20 ± 0.20)^1c^	30.80 ± 1.31^3c^ (32.80 ± 1.88)^3c^	20.40 ± 0.50^2c^ (22.40 ± 1.74)^3^
ASD‐760	2.65 ± 0.20^3^ (0.88 ± 0.12)	3.20 ± 0.20 (1.80 ± 0.20)^3c^	34.40 ± 0.50^3^ (23.20 ± 1.82)^c^	22.00 ± 1.04^1c^ (22.40 ± 1.47)^c^
NT‐190	1.54 ± 0.18 (2.28 ± 0.17)^c^	3.00 ± 0.44 (3.60 ± 0.24)	30.00 ± 0.31^c^ (35.20 ± 1.74)^c^	12.00 ± 0.44 (22.40 ± 1.74)^c^

*Note*: Each value represents the mean ± MSE; *n* = 5. ^a^ *p* < 0.05; ^b^ *p* < 0.01; ^c^ *p* < 0.001: significant differences versus NTC. ^1^ *p* < 0.05; ^2^ *p* < 0.01; ^3^ *p* < 0.001: significant differences versus ASD. NTC: neurotypical animals treated with distilled water (10 mL/kg) ASD: ASD animal treated with distilled water (10 mL/kg); ASD‐B: ASD animal treated at bumetanide (4 mg/kg); ASD‐190, ASD‐760: ASD animals treated with the aqueous extract *P. africanum* at doses of 190 and 760 mg/kg; NT‐190: neurotypical animal treated with the aqueous extract from *P. africanum* at dose of 190 mg/kg. Wave 1 (starting 28 days after weaning) is represented by data outside brackets. Wave 2 (starting 37 days after weaning) is represented by data in brackets.

The study found that after weaning, ASD rats treated with *P. africanum* at 190 mg/kg showed a 31.62% increase, while those given the 760 mg/kg extract dose showed a 47% increase in the number of lines crossed. Bumetanide also showed a 53.75% increase compared to the negative control. The administration of these doses increased exploration by 34.66%, 53.78%, and 38.64%, respectively, compared to NTC rats. These results were consistent in the second wave, with the 190 mg/kg dose of extract and Bumetanide showing increases of 59.22% (*p* < 0.001) and 49.51% (*p* < 0.01), respectively, compared to the NTC animals.

Data from the OA are consistent with VPA's on stereotyped behavior. Indeed, the number of groomings in the open arena increased in the negative control compared with the normal by 56.93% (*p* < 0.001) in wave 1 and 52.85% (*p* < 0.001) in wave 2. Treatment with extract at doses of 190 and 760 mg/kg and with bumetanide decreased the number of groomings compared with the negative control respectively by 24.54% (*p* < 0.01), 19.70% (*p* < 0.05), and 32.11% (*p* < 0.001) in wave 1 and by 72.88% (*p* < 0.001), 86.44% (*p* < 0.001), and 57.62% (*p* < 0.01) compared with the NTC rats. Compared to the ASD group in the second wave, the number of groomings was reduced by 36.42% (*p* < 0.001) for the 190 mg/kg dose of extract, by 22.85% (*p* < 0.05) for bumetanide and by 69.69% (*p* < 0.001) for the 760 mg/kg dose of extract.

#### 3.6.4 Effects on Exploratory Activity and Anxiety in the Elevated plus Maze

Data from the elevated plus maze test (EPM), a standard screening for putative anxiolytic compounds, are resumed in Table 8. In the study, the time spent in open arms decreased in the ASD group compared to NTC animals by 92.41% and 96.24% in waves 1 and 2, respectively. However, extract doses of 190 and 760 mg/kg significantly increased the time spent in open arms. Bumetanide also increased the time spent in open arms in both waves. In the NTC group, the time spent in open arms increased significantly compared to the neurotypical control during wave 1. Furthermore, the ASD group had 80% fewer open‐arm entries than the normal control group. However, the group that received an extract of 190 mg/kg had a 2.5‐fold increase in open arm entries in wave 1 and an 83% increase in wave 2 compared to the negative control group. The 760 mg/kg dose of extract showed even more promising results. It increased the number of open‐arm entries by 3‐fold in wave 1 and by 80% in wave 2 compared to the negative control group. Bumetanide increased the number of entries into the open arms by 85.71% in wave 1 compared to the negative control group. The administration of *P. africanum* at 190 mg/kg showed a significant decrease in time spent in the closed arms. In wave 1, there was a 23.77% decrease compared to the ASD group and a 25.05% decrease for the 760 mg/kg extract dose. In wave 2, the time spent in the closed arms was reduced by 8.84% for the 190 mg/kg dose of extract and by 7.63% for bumetanide compared to the negative control group. The group exposed to sodium valproate in utero had significantly more entries into the closed arms than the NTC. However, the treatment per os with *P. africanum* at 190 mg/kg reduced this number by 58.33% for wave 2 compared to ASD rats.

**TABLE 8:** Effects of *Piptadeniastrum africanum* aqueous extract on exploratory activity and anxiety in the elevated cross‐maze.

Groups	Time spent in the OA(s)	Number of entries in the OA	Time spent in the CA (s)	Number of entries in the CA	Number of regressions	Number of headfalls	Number of groomings
NTC	10.55 ± 0.25 (5.33 ± 0.18)	1.00 ± 0.00 (1.00 ± 0.00)	267.60 ± 5.66 (272.00 ± 2.93)	1.80 ± 0.20 (1.40 ± 0.24)	2.40 ± 0.24 (2.40 ± 0.24)	1.00 ± 0.00 (0.80 ± 0.20)	17.40 ± 0.40 (25.00 ± 1.97)
ASD	0.80 ± 0.20^c^ (0.20 ± 0.20)^c^	0.20 ± 0.20^a^ (0.20 ± 0.20)^a^	290.70 ± 2.18 (292.30 ± 2.22)^a^	3.20 ± 0.20^b^ (2.40 ± 0.24)^a^	4.80 ± 0.37^c^ (6.40 ± 0.24)^c^	0.40 ± 0.24 (0.00 ± 0.00)	57.00 ± 0.83^c^ (53.60 ± 2.01)^c^
ASD‐B	7.00 ± 0.31^3c^ (9.16 ± 0.70)^3c^	1.40 ± 0.24^2^ (0.80 ± 0.20)	277.00 ± 4.75 (270.00 ± 2.58)^2^	2.00 ± 0.31^1^ (2.40 ± 0.24)^a^	4.80 ± 0.37^c^ (4.40 ± 0.24)^3c^	0.60 ± 0.24 (0.80 ± 0.37)	16.80 ± 0.37^3^ (28.40 ± 1.20)^3^
ASD‐190	15.12 ± 0.36^3c^ (11.55 ± 0.42)^3c^	2.00 ± 0.00^3b^ (1.20 ± 0.20)^2^	221.60 ± 10.17^3b^ (266.50 ± 7.78)^2^	2.60 ± 0.24 (1.00 ± 0.00)^2^	2.00 ± 0.31^3^ (5.00 ± 0.31)^1c^	1.40 ± 0.24^1^ (1.60 ± 0.40)^2^	38.40 ± 2.06^3c^ (36.40 ± 1.16) ^3c^
ASD‐760	20.57 ± 0.26^3c^ (11.80 ± 0.37)^3c^	2.20 ± 0.20^3b^ (1.00 ± 0.00)^1^	217.90 ± 9.34^3b^ (275.80 ± 3.38)	1.40 ± 0.24^3^ (1.60 ± 0.24)	0.80 ± 0.37^3a^ (1.00 ± 0.31)^3a^	1.40 ± 0.24^1^ (2.20 ± 0.20)^3a^	45.40 ± 2.31^3c^ (40.80 ± 2.17)^3c^
NT‐190	18.87 ± 0.42^c^ (5.45 ± 0.24)	1.40 ± 0.24 (0.80 ± 0.20)	239.30 ± 12.15 (280.70 ± 1.00)	2.00 ± 0.31 (1.80 ± 0.20)	2.40 ± 0.24 (3.60 ± 0.24)^a^	2.40 ± 0.24^b^ (0.40 ± 0.24)	48.40 ± 1.93^c^ (49.60 ± 1.16)^c^

*Note*: Each value represents the mean ± MSE; *n* = 5. ^a^ *p* < 0.05; ^b^ *p* < 0.01; ^c^ *p* < 0.001: significant differences versus NTC. ^1^ *p* < 0.05; ^2^ *p* < 0.01; ^3^ *p* < 0.001: significant differences versus ASD. OA: opened arms; CA: closed arms; NTC: neurotypical animals treated with distilled water (10 mL/kg) ASD: ASD animal treated with distilled water (10 mL/kg); ASD‐B: ASD animal treated at bumetanide (4 mg/kg); ASD‐190, ASD‐760: ASD animals treated with the aqueous extract *P. africanum* at doses of 190 and 760 mg/kg; NT‐190: neurotypical animal treated with the aqueous extract from *P. africanum* at the dose of 190 mg/kg. Wave 1 (starting 28 days after weaning) is represented by data outside brackets. Wave 2 (starting 37 days after weaning) is represented by data in brackets.

During the experiment, it was found that the number of movements in the elevated cross significantly increased in the negative control compared to NTC. However, treatment with the extract at doses of 190 and 760 mg/kg and bumetanide reduced the number of movements. In addition, the number of head falls increased in the pharmacological control, but *P. africanum* and Bumetanide restored the number of grooming activities to average values. The number of sit‐ups and groomings increased in the pharmacological control compared to NTC. The study found that treatment with the *P. africanum* extract at all doses reduced the number of movements in the elevated cross by 58.33% and 83.33%, respectively, compared to the negative control. Bumetanide also reduced the number of movements in the second wave. The number of head falls increased in the pharmacological control group compared to the neurotypical rats in the first wave but was restored to standard value by *P. africanum* and bumetanide. The number of sit‐ups increased in the pharmacological control group compared to NTC in the second wave. Additionally, the number of groomings in the pharmacological control group increased compared to NTC in both waves.

#### 3.6.5 Effects on Pain Sensitivity During the Hot Plate Test

**FIGURE 5:**
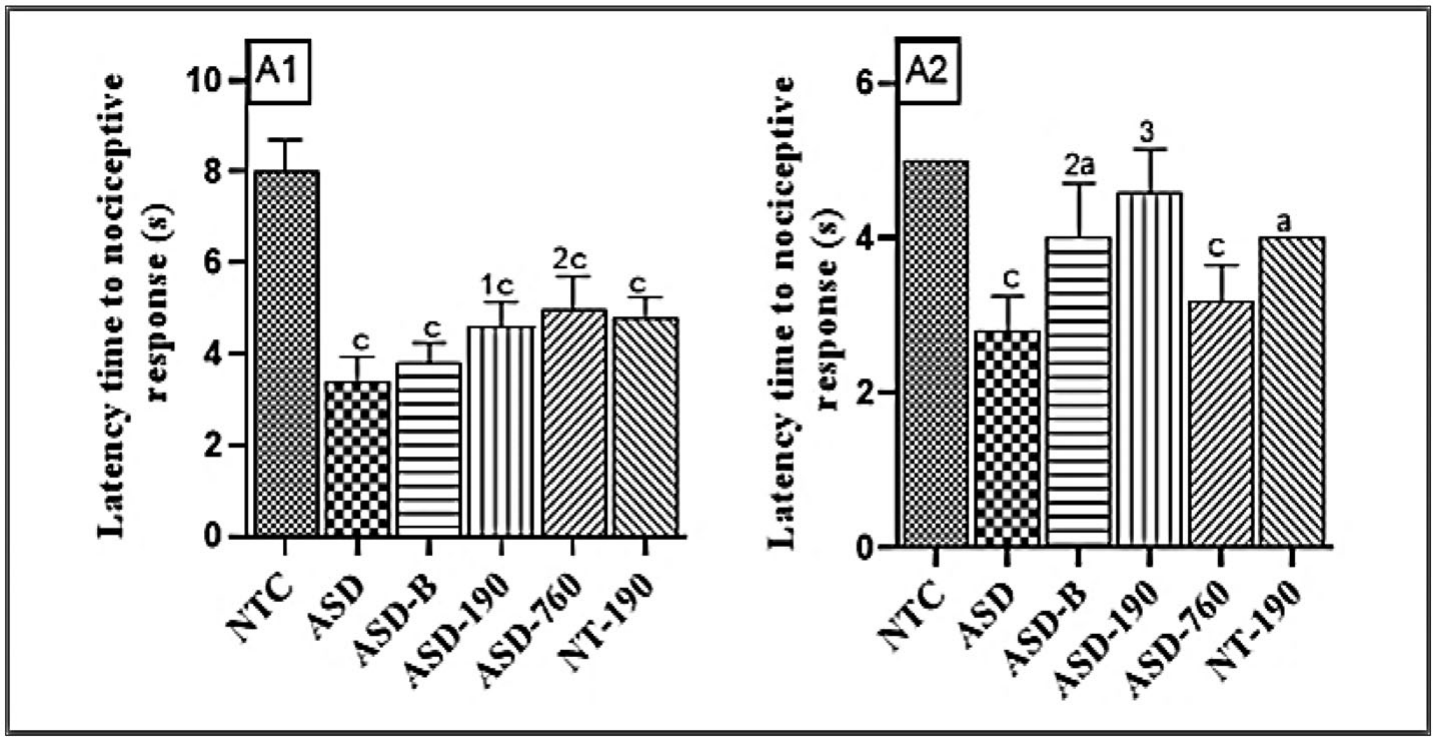
Effects of *Piptadeniastrum africanum* aqueous extract on pain sensitivity during the hot plate test. Each bar represents the mean ± MSE; *n* = 5. ^a^ *p* < 0.05; ^c^ *p* < 0.001: significant differences versus NTC. ^1^ *p* < 0.05; ^2^ *p* < 0.01; ^3^ *p* < 0.001: significant differences versus ASD. NTC: neurotypical animals treated with distilled water (10 mL/kg); ASD: ASD animal treated with distilled water (10 mL/kg); ASD‐B: ASD animal treated at Bumetanide (4 mg/kg); ASD‐190, ASD‐760: ASD animals treated with the aqueous extract *P. africanum* at doses of 190 and 760 mg/kg; NT‐190: neurotypical animal treated with the aqueous extract from *P. africanum* at the dose of 190 mg/kg. A1 and A2 designate waves 1 and 2, respectively.

The administration of sodium valproate (VPA) during pregnancy was observed to reduce the time it took for the offspring to respond to pain stimuli 28 and 37 days after weaning (Figure 5). The reduction was 57.5% (Mean: 3.40; SD: 0.54) (wave 1) and 44% (Mean: 2.80; SD: 0.44) (wave 2) compared to the normal control (NTC) on the hot plate. However, doses of 190 and 760 mg/kg of the extract increased the response time by 35% (Mean: 4.60; SD: 0.54) and 47.05% (Mean: 5.00; SD: 0.70), respectively, for wave 1. For wave 2, the 190 mg/kg extract and Bumetanide increased the latency time by 64.28% (Mean: 4.60; SD: 0.54) and 42.28% (Mean: 4.00; SD: 0.70), respectively, compared to the negative control. The pharmacological control decreased by 40% (wave 1) and 20% (wave 2) compared to neurotypical rats. *P. africanum* at 190 and 760 mg/kg, as well as Bumetanide, increased the response time compared to the NTC group. This increase was 45.50% and 37.50% for 190 and 760 mg/kg doses of extract, respectively, and 52.50% for Bumetanide in wave 1.

### 3.7 Effects on some Biochemical Parameters

#### 3.7.1 Effects on GABA Levels

**FIGURE 6:**
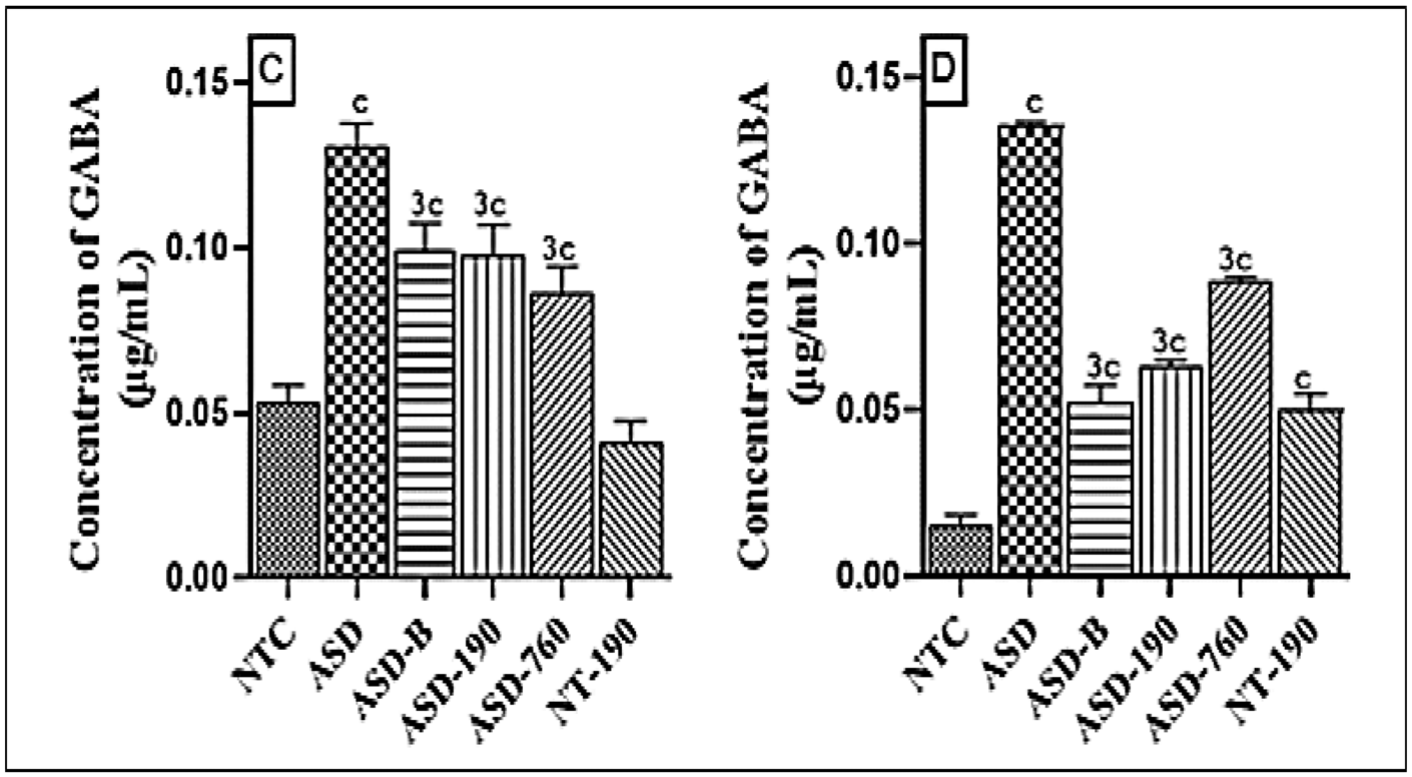
Effects of *Piptadeniastrum africanum* aqueous extract on GABA concentration in hippocampus (C), and amygdala (D). Each bar represents the mean ± MSE; *n* = 5. ^c^ *p* < 0.001: significant differences versus NTC. ^3^ *p* < 0.001: significant differences versus ASD. NTC: neurotypical animals treated with distilled water (10 mL/kg); ASD: ASD animal treated with distilled water (10 mL/kg); ASD‐B: ASD animal treated at Bumetanide (4 mg/kg); ASD‐190, ASD‐760: ASD animals treated with the aqueous extract *P. africanum* at doses of 190 and 760 mg/kg; NT‐190: neurotypical animal treated with the aqueous extract from *P. africanum* at dose of 190 mg/kg.

During gestation days 11, 12, and 13, female animals treated with sodium valproate exhibited a significant increase in GABA concentration in various brain areas compared to ASD control (Figure 6). This increase was approximately 2.5‐fold (*p <* 0,001; Mean: 0.13; SD: 0.00) in the hippocampus and 5‐fold (*p* < 0.001; Mean: 0.13; SD: 0) in the amygdala. However, the pharmacological control showed increased GABA concentration in the hippocampus and amygdala compared to the neurotypical animals. Furthermore, the extract corrected the increased GABA concentration in the hippocampus and amygdala. The concentration of GABA decreased in all areas of the brain in animals treated with 190 mg/kg of the extract compared to the negative control. Similarly, the concentration of GABA decreased in the hippocampus and amygdala in animals treated with 760 mg/kg of the extract. Bumetanide also caused a decrease in GABA concentration in all areas of the brain compared to the neurotypical rats.

#### 3.7.2 Effects on GABA‐T Activity

**FIGURE 7:**
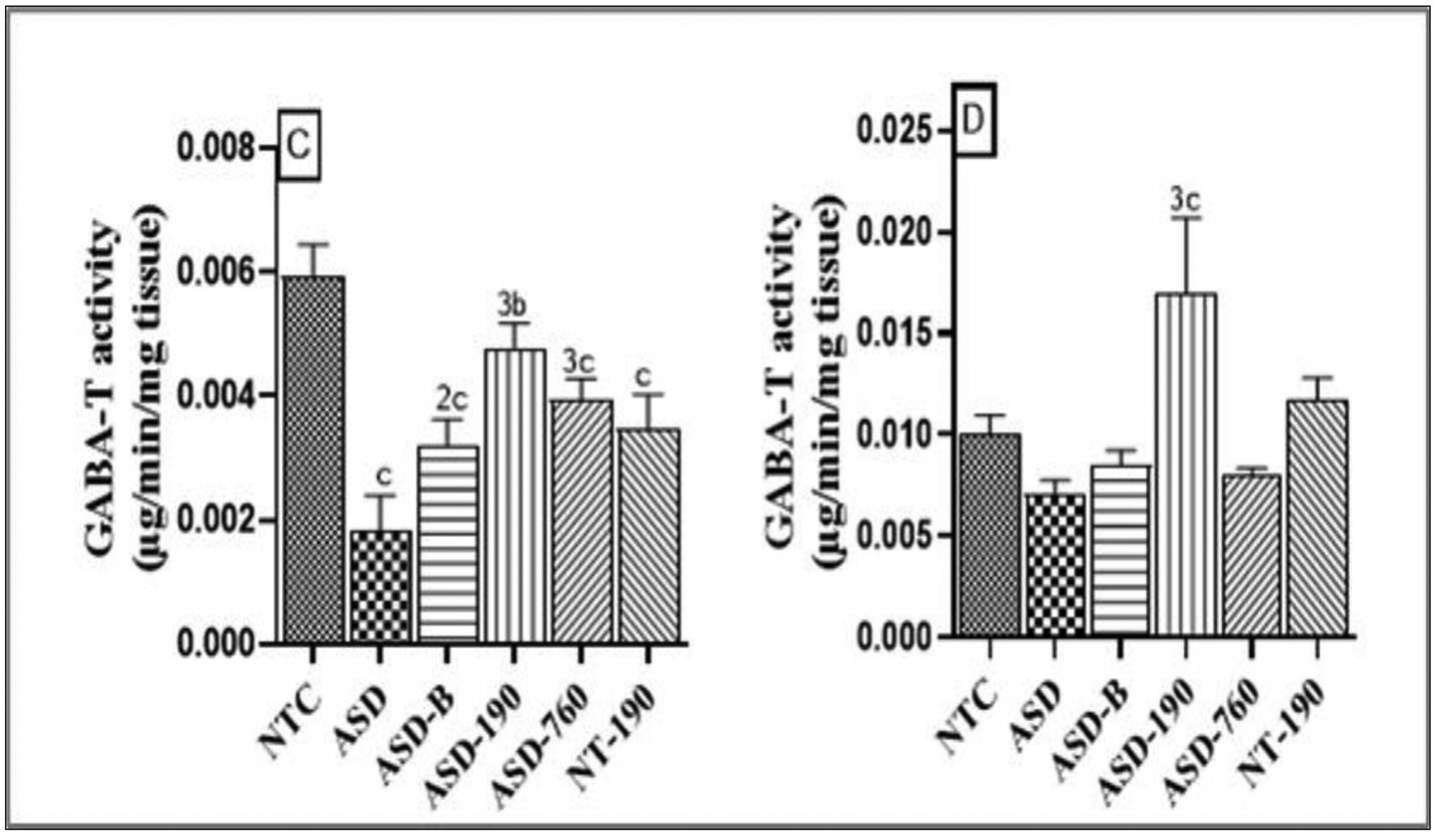
Effects of *Piptadeniastrum africanum* aqueous extract on GABA‐T activity in the hippocampus (C) and amygdala (D). Each bar represents the mean ± MSE; *n* = 5. ^b^ *p* < 0.01; ^c^ *p* < 0.001: significant differences versus NTC. ^2^ *p* < 0.01; ^3^ *p* < 0.001: significant differences versus ASD. NTC: neurotypical animals treated with distilled water (10 mL/kg); ASD: ASD animal treated with distilled water (10 mL/kg); ASD‐B: ASD animal treated at Bumetanide (4 mg/kg); ASD‐190, ASD‐760: ASD animals treated with the aqueous extract *P. africanum* at doses of 190 and 760 mg/kg; NT‐190: neurotypical animal treated with the aqueous extract from *P. africanum* at dose of 190 mg/kg.

GABA‐T activity in the hippocampus (Figure 7C) decreased by 69.02% (*p* < 0.001; Mean: 0.001; SD: 0.000) in ASD control compared to neurotypical animals after sodium valproate administration to pregnant females. The decrease in pharmacological control was 41.41% (*p* < 0.001) compared to the NTC group after birth. However, treatment with *P. africanum* extract and bumetanide corrected this decrease, increasing by 60.66% (*p* < 0.001; Mean: 0.004; SD: 0.000) for the 190 mg/kg dose, 53.84% (*p* < 0.001; Mean: 0.003; SD: 0.000) for the 760 mg/kg dose of extract, and 42.85% (*p* < 0.01; Mean: 0.003; SD: 0.000) for Bumetanide in ASD animals compared to the vehicle control. In the amygdala (Figure 7D), there was a 2.5‐fold (*p* < 0.001; Mean: 0.016; SD: 0.003) increase in GABA‐T activity due to the 190 mg/kg dose compared with the negative control and a 67.92% (*p* < 0.001) increase compared with the NTC rats. Compared with the neurotypical rats, GABA‐T activity in the hippocampus was increased by 19.52% (*p* < 0.01), 33.67% (*p* < 0.001) for 190 and 760 mg/kg extract and 45.79% (*p* < 0.001) for bumetanide.

#### 3.7.3 Effects on L‐GAD Activity

**FIGURE 8:**
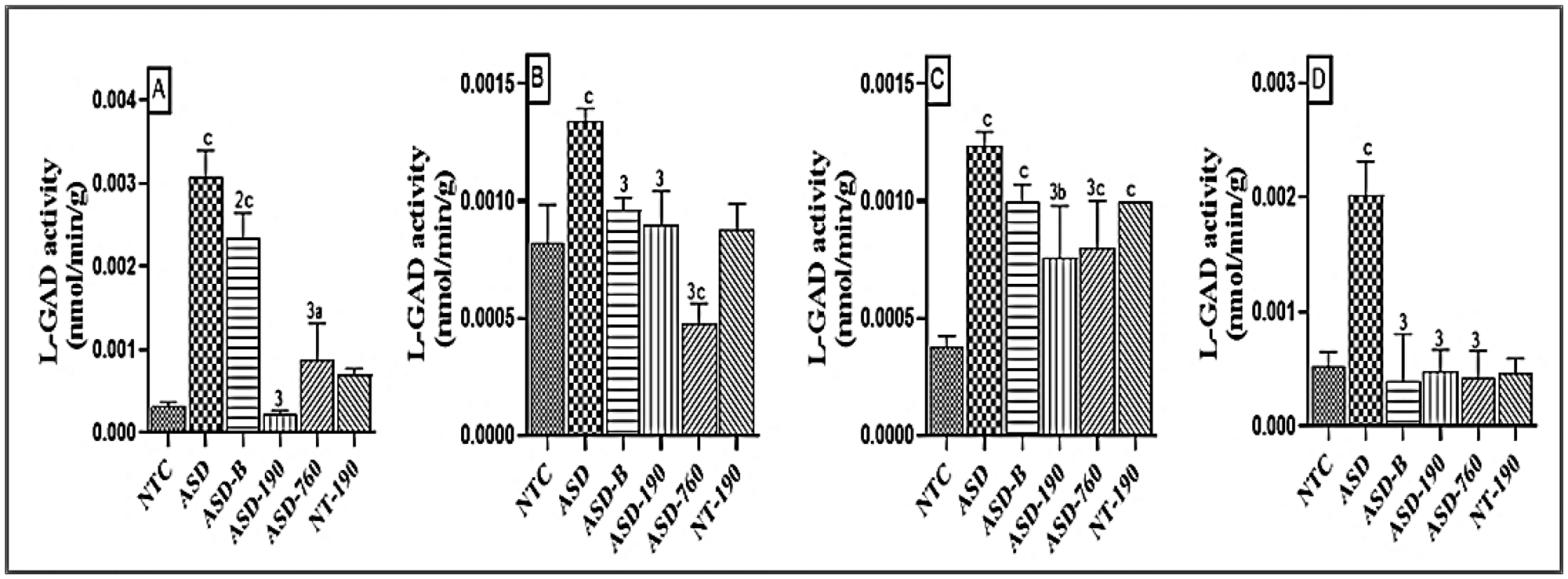
Effects of *Piptadeniastrum africanum* aqueous extract on L‐GAD activity in the cerebellum (A), prefrontal cortex (B), hippocampus (C), and amygdala (D). Each bar represents the mean ± MSE; *n* = 5. ^a^ *p* < 0.05; ^b^ *p* < 0.01; ^c^ *p* < 0.001: significant differences versus NTC. ^2^ *p* < 0.01; ^3^ *p* < 0.001: significant differences versus ASD. NTC: neurotypical animals treated with distilled water (10 mL/kg); ASD: ASD animal treated with distilled water (10 mL/kg); ASD‐B: ASD animal treated at Bumetanide (4 mg/kg); ASD‐190, ASD‐760: ASD animals treated with the aqueous extract *P. africanum* at doses of 190 and 760 mg/kg; NT‐190: neurotypical animal treated with the aqueous extract from *P. africanum* at dose of 190 mg/kg.

An increase in L‐GAD activity was observed in the ASD animals in the cerebellum (Figure 8A), prefrontal cortex (Figure 8B), hippocampus (Figure 8C), and amygdala (Figure 8D). Compared with neurotypical rats, this increase was 8‐fold (*p* < 0.001; Mean: 0.0030; SD: 0.0003), 63.41% (*p* < 0.001), 3‐fold (*p* < 0.001), and 2.5‐fold (*p* < 0.001), respectively. Treatment with *P. africanum* extract at a daily dose of 190 mg/kg significantly reduced L‐GAD activity, with reductions of 92.85% (*p* < 0.001) in the cerebellum, 32.83% (*p* < 0.001) in the prefrontal cortex, 38.70% (*p* < 0.001) in the hippocampus, and 76.25% (*p* < 0.001) in the amygdala compared to the NTC. The impact of the plant extract on L‐GAD activity was more pronounced at 760 mg/kg, with reductions of 63.63% (*p* < 0.05; Mean: 0.0008; SD: 0.0004) in the cerebellum, 41.46% (*p* < 0.001; Mean: 0.0001; SD: 0.0000) in the prefrontal cortex and 52.50% (*p* < 0.001) in the hippocampus, compared to the ASD control. Bumetanide (4 mg/kg) significantly decreased L‐GAD activity in ASD rats by 86.30% (*p* < 0.001) in the cerebellum and 62% (*p* < 0.001) in the hippocampus.

#### 3.7.4 Effects on Serotonin Levels

**FIGURE 9:**
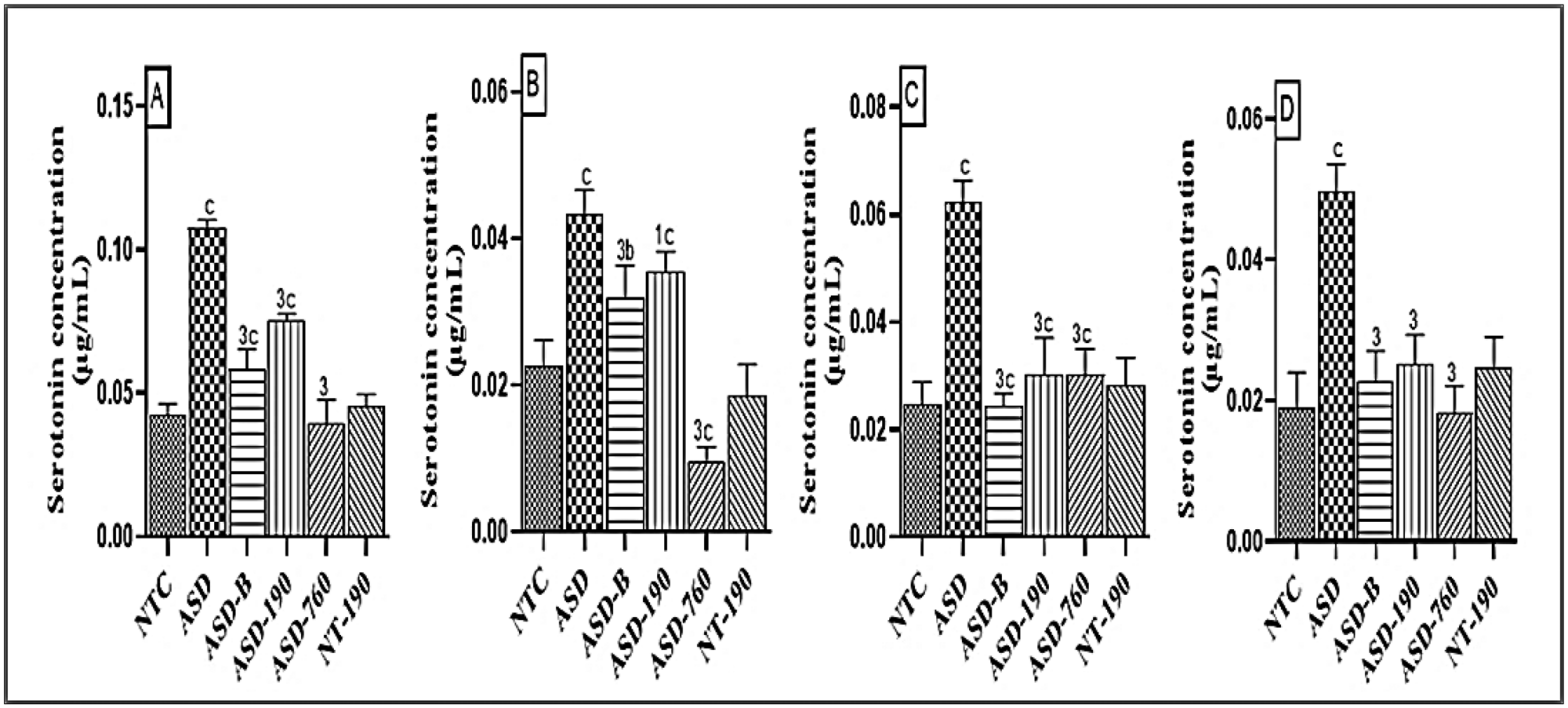
Effects of *Piptadeniastrum africanum* aqueous extract on serotonin concentration in the cerebellum (A), prefrontal cortex (B), hippocampus (C), and amygdala (D). Each bar represents the mean ± MSE; *n* = 5. ^b^ *p <* 0.01; ^c^ *p <* 0.001: significant differences from normal control. ^1^ *p <* 0.05; ^3^ *p <* 0.001: significant differences from negative control. NTC: neurotypical animals treated with distilled water (10 mL/kg) ASD: ASD animal treated with distilled water (10 mL/kg); ASD‐B: ASD animal treated at Bumetanide (4 mg/kg); ASD‐190, ASD‐760: ASD animals treated with the aqueous extract *P. africanum* at doses of 190 and 760 mg/kg; NT‐190: neurotypical animal treated with the aqueous extract from *P. africanum* at dose of 190 mg/kg.

Serotonin (5‐HT) concentration was significantly increased in ASD animals in the various brain regions considered. Compared with the neurotypical rats, there was an increase of 60.22% (*p* < 0.001; Mean: 0.107; SD: 0.002), 90.07% (p < 0.001; Mean: 0.043; SD: 0.003), 60.19% (*p <* 0.001; Mean: 0.062; SD: 0.004), and 61.84% (*p <* 0.001; Mean: 0.049; SD: 0.003), respectively, in the cerebellum (Figure 9A), prefrontal cortex (Figure 9B), hippocampus (Figure 9C), and amygdala (Figure 9D). Treatment with different doses of the extract and bumetanide resulted in a significant drop in serotonin levels. At 190 mg/kg, compared with the ASD control, *P. africanum* reduced 5‐HT levels by 30.04% (*p <* 0.001; Mean: 0.058; SD: 0.006), 17.78% (*p <* 0.05; Mean: 0.035; SD: 0.002), 51.18% (*p <* 0.001; Mean: 0.030; SD: 0.006), and 49.21% (*p <* 0.001; Mean: 0.025; SD: 0.004). Bumetanide induced a likewise decrease of 5‐HT concentrations by 45.75% (*p <* 0.001), 26% (*p <* 0.001) in the cerebellum and prefrontal cortex, respectively, 60.64% (*p <* 0.001) in the hippocampus and 54.12% (*p <* 0.001) in the amygdala compared with the ASD control.

### 3.8 Effects on Oxidative Stress Markers

#### 3.8.1 Effects on Reduced Glutathione Concentration

**FIGURE 10:**
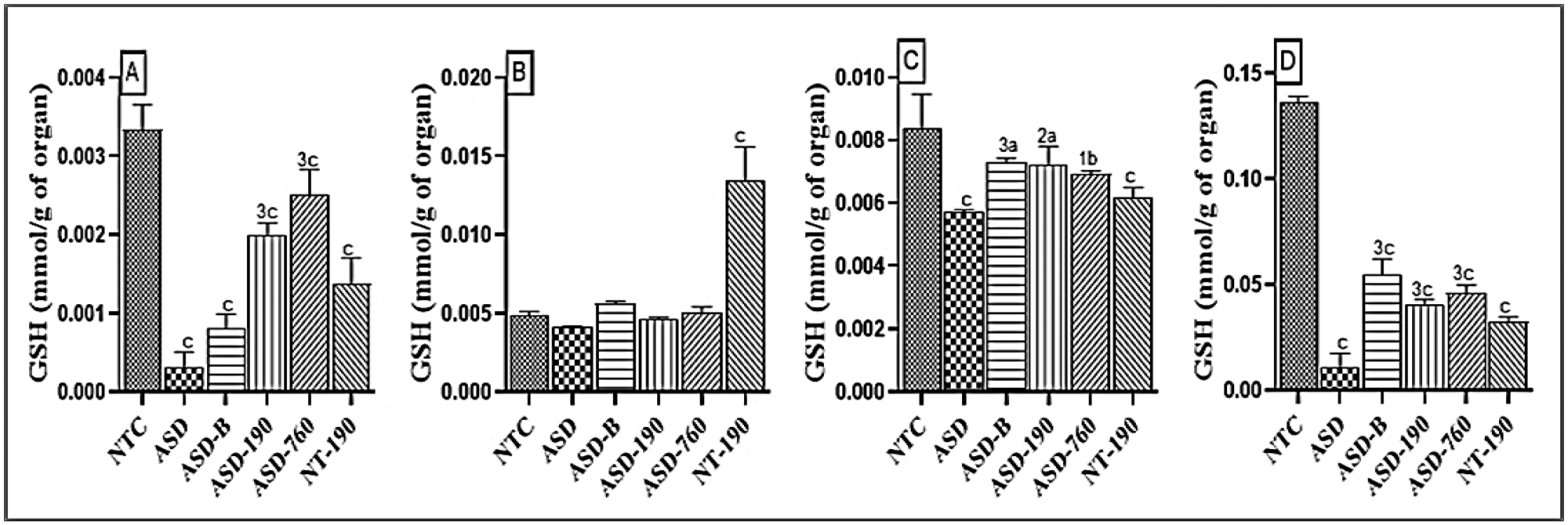
Effects of *Piptadeniastrum africanum* aqueous extract on reduced glutathione concentration in the cerebellum (A), prefrontal cortex (B), hippocampus (C), and amygdala (D). Each bar represents the mean ± MSE; *n* = 5. ^a^ *p <* 0.05; ^b^ *p <* 0.01; ^c^ *p <* 0.001: significant differences versus NTC. ^1^ *p <* 0.05; ^2^ *p <* 0.01; ^3^ *p <* 0.001: significant differences versus ASD. NTC: neurotypical animals treated with distilled water (10 mL/kg); ASD: ASD animal treated with distilled water (10 mL/kg); ASD‐B: ASD animal treated at Bumetanide (4 mg/kg); ASD‐190, ASD‐760: ASD animals treated with the aqueous extract *P. africanum* at doses of 190 and 760 mg/kg; NT‐190: neurotypical animal treated with the aqueous extract from *P. africanum* at dose of 190 mg/kg.

Impairment of glutathione function in the brain is linked to the loss of neurons during aging or as a result of neurological diseases. As shown in Figure 10, sodium valproate administration to females decreased the GSH concentration in offspring. In the ASD rats, the extract at doses of 190 and 760 mg/kg increased GSH concentration in the cerebellum by 4‐fold (Mean: 0.002; SD: 0.000) and 5‐fold (Mean: 0.002; SD: 0.000), respectively. In animals treated with *P. africanum* extract, GSH concentration increased by 40.11% (*p <* 0.001) in the cerebellum, 13.41% (*p <* 0.01) in the hippocampus, and 70.36% (*p <* 0.001) in the amygdala, compared to the neurotypical animals. Bumetanide also increased GSH concentration in various regions of the brain.

#### 3.8.2 Effects on Malondialdehyde Levels

**FIGURE 11:**
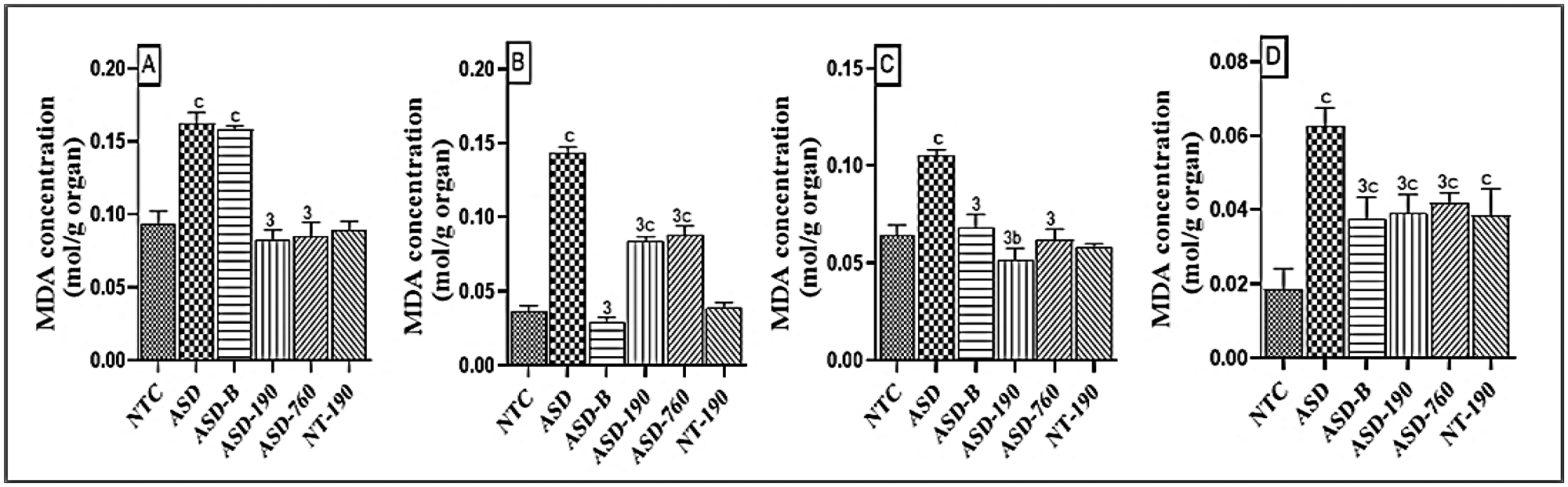
Effects of *Piptadeniastrum africanum* aqueous extract on malondialdehyde concentration in the cerebellum (A), prefrontal cortex (B), hippocampus (C), and amygdala (D). Each bar represents the mean ± MSE; *n* = 5. ^b^ *p <* 0.01; ^c^ *p <* 0.001: significant differences versus NTC. ^3^ *p <* 0.001: significant difference versus ASD. NTC: neurotypical animals treated with distilled water (10 mL/kg) ASD: ASD animal treated with distilled water (10 mL/kg); ASD‐B: ASD animal treated at Bumetanide (4 mg/kg); ASD‐190, ASD‐760: ASD animals treated with the aqueous extract *P. africanum* at doses of 190 and 760 mg/kg; NT‐190: neurotypical animal treated with the aqueous extract from *P. africanum* at a dose of 190 mg/kg.

Malondialdehyde (MDA) is a marker of oxidative stress and one of the cells' final products of polyunsaturated fatty acids peroxidation. MDA concentration increased in the negative control group's cerebellum, prefrontal cortex, hippocampus, and amygdala (Figure 11). Compared to the neurotypical control group, MDA levels increased by 73.45% (*p <* 0.001) in the cerebellum and hippocampus, and 2.5‐fold in the prefrontal cortex and amygdala in the offspring of female VPA‐treated. The means are 0.162 (SD: 0.007), 0.105 (SD: 0.002), 0.143 (SD: 0.003), 0.062 (SD: 0.005), respectively. The NT‐190 control group also showed an increase in MDA concentration. The administration of the extract at a dose of 190 and 760 mg/kg decreased the MDA concentration in the cerebellum, prefrontal cortex, hippocampus, and amygdala, compared to the negative control group (*p <* 0.001). The Bumetanide‐treated group observed decreased MDA concentration in the prefrontal cortex, hippocampus, and amygdala compared to the negative control group (*p <* 0.001). However, MDA concentration after Bumetanide administration increased in the cerebellum and amygdala compared to the neurotypical control group. The extract's 190 mg/kg dose increased MDA concentration in the prefrontal cortex, hippocampus, and amygdala compared to the NTC rats (*p <* 0.001).

#### 3.8.3 Effects on Superoxide Dismutase Activity

**FIGURE 12:**
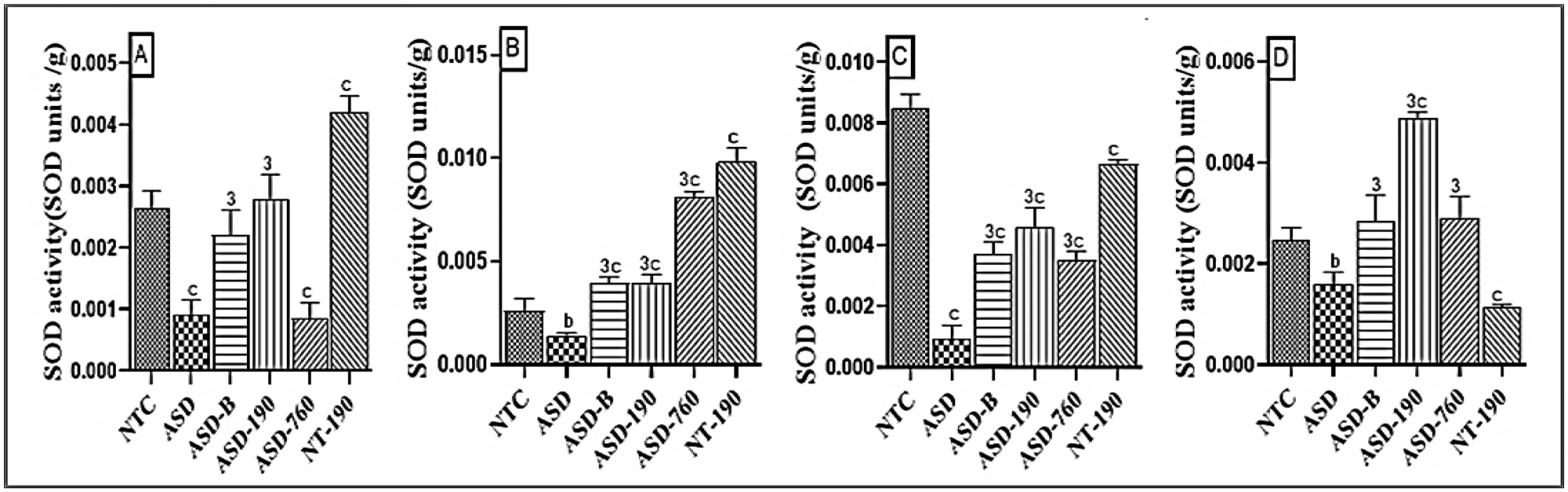
Effects of *Piptadeniastrum africanum* aqueous extract on superoxide dismutase activity in cerebellum (A), prefrontal cortex (B), hippocampus (C), and amygdala (D). Each bar represents the mean ± MSE; *n* = 5. ^b^ *p <* 0.01; ^c^ *p <* 0.001: significant differences versus NTC. ^3^ *p <* 0.001: significant differences versus ASD. NTC: neurotypical animals treated with distilled water (10 mL/kg) ASD: ASD animal treated with distilled water (10 mL/kg); ASD‐B: ASD animal treated at Bumetanide (4 mg/kg); ASD‐190, ASD‐760: ASD animals treated with the aqueous extract *P. africanum* at doses of 190 and 760 mg/kg; NT‐190: neurotypical animal treated with the aqueous extract from *P. africanum* at a dose of 190 mg/kg.

SOD constitutes a critical antioxidant defense against oxidative stress in the body and catalyzes the dismutation of superoxide anion free radical (O^2−^) into molecular oxygen and hydrogen peroxide (H_2_O_2_). SOD activity significantly decreased in the negative control group compared to the neurotypical animals. As shown in Figure 12, there was a decrease of 65.53% (Mean: 0.0009; SD: 0.0002) in the cerebellum; in the prefrontal cortex, there was a decrease of 46.10% (Mean: 0.0014; SD: 0.0001); in the hippocampus, there was a decrease of 88.91% (Mean: 0.0004; SD: 0.0009); and in the amygdala, there was a decrease of 35.77% (Mean: 0.0015; SD: 0.0002). The 190 mg/kg extract and Bumetanide increased SOD activity in the cerebellum, prefrontal cortex, hippocampus, and amygdala compared to the negative control. SOD activity doubled in animals treated with 190 mg/kg of plant extract compared to the NTC group. Compared with the NTC group, SOD activity in the group of rats treated with 760 mg/kg of the extract increased in the cerebellum by 67.80% (*p <* 0.0001), in the prefrontal cortex by 67.89% (*p <* 0.001), and in the hippocampus by 58.72% (*p <* 0.001). In the same direction, compared with neurotypical rats, Bumetanide and the 190 mg/kg dose promoted an increase of 51.14% (*p <* 0.001) in the prefrontal cortex. This increase from the NTC group was 56.36% (*p <* 0.001) for the group treated at a dose of 190 mg/kg and 45.75% (*p <* 0.001) for the group treated with Bumetanide in the amygdala.

### 3.9 Effects on the Microarchitecture of the Cerebellum, Hippocampus, and Amygdala

**FIGURE 13:**
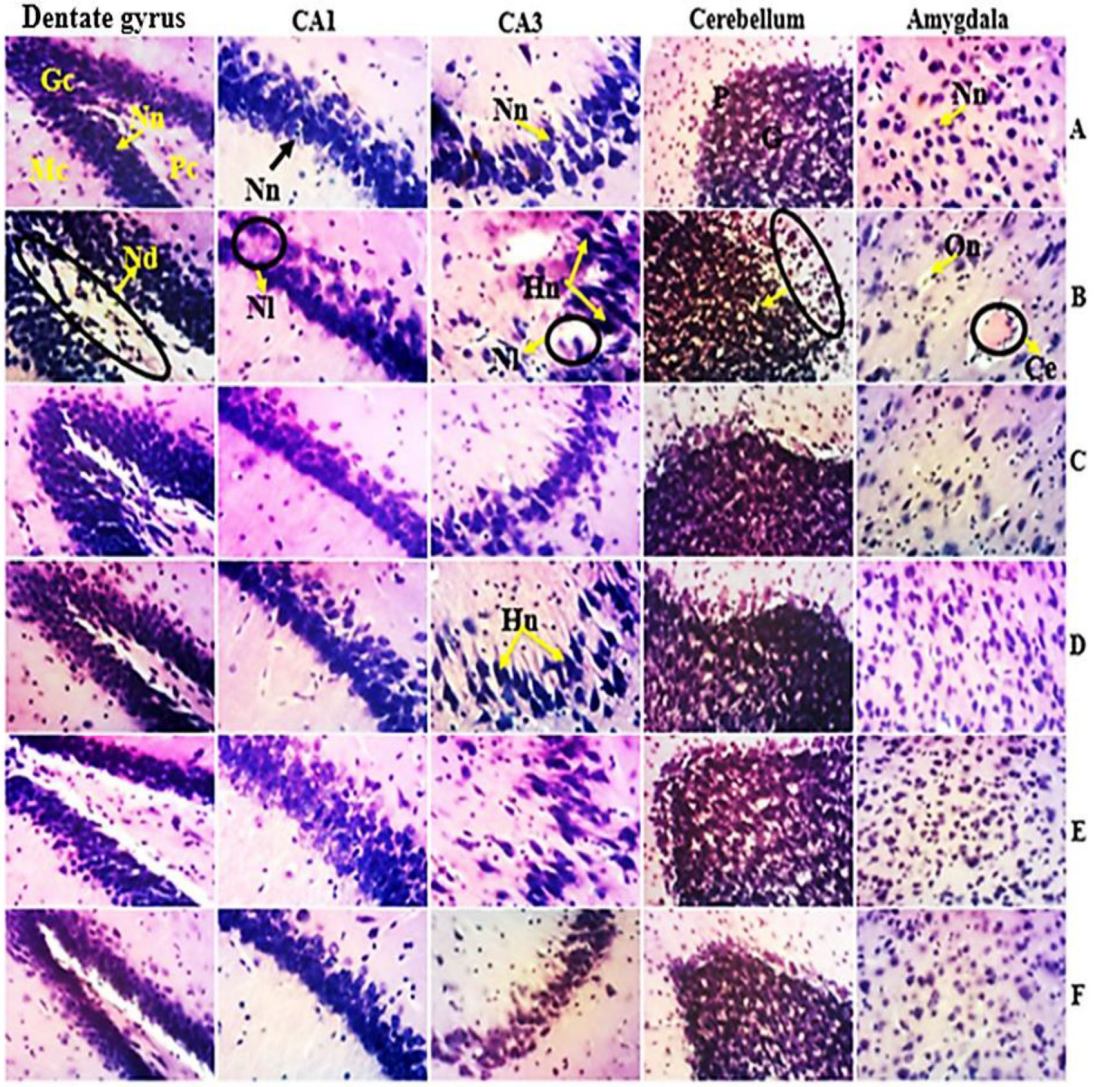
Microphotographs of the microarchitecture of the cerebellum, amygdala, and hippocampus (200×, H&E). A = neurotypical animals receiving distilled water (10 mL/kg); B = ASD animals receiving distilled water (10 mL/kg); C = ASD animals receiving reference drug. D, E = ASD animals receiving the aqueous extract of *P. africanum* at 190 and 760 mg/kg; F = neurotypical animals receiving the aqueous extract of *P. africanum* at 190 mg/kg. CA1 and CA3: Ammon horn regions 1 and 2; Gc = granular cell layer; Mc = molecular layer; Pc = polymorphic cell layer; Nd = neuronal degeneration; Ce = cerebral edema; G = granular cell layer; Hn = hyperchromatic nucleus; Nn = normal neuron; On = oligodendrocyte necrosis; P = Purkinje cell layer; Nl = neuronal loss.

After conducting histological analysis, it was confirmed that administering sodium valproate to pregnant females has harmful effects on the neurons, oligodendrocytes, and neuron organization in the brains of their offspring. According to Figure 13, the H&E staining revealed that rats with ASD showed increased hyperchromatic and vacuolated neurons in the pyramidal cell layer in both CA1 and CA3 of the hippocampus. Furthermore, administering VPA to pregnant females also resulted in oligodendrocyte necrosis associated with cerebral edema in the amygdala, decreased the number of neurons in the Purkinje cell layer, and neuronal degeneration in the dentate gyrus. The dentate gyrus, responsible for emotion and memory, is known to be involved in autism spectrum disorders. Compared with neurotypical rats, the aqueous extract of *P. africanum* and bumetanide attenuated these alterations.

### 3.10 Effects on Hippocampal and Amygdala Neuron Counting

**FIGURE 14:**
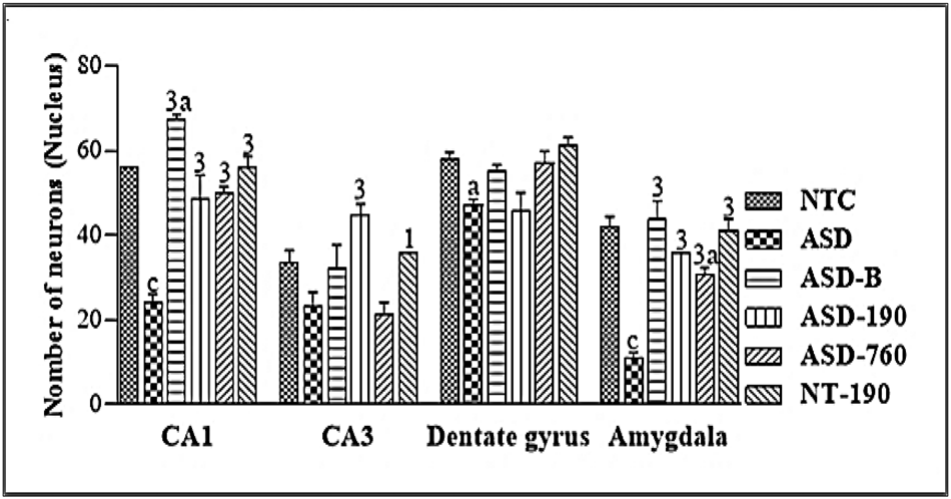
Effects of *Piptadeniastrum africanum* aqueous extract on neuron counting in the hippocampus and amygdala. Each bar represents the mean ± MSE; *n* = 5. ^a^ *p <* 0.05; ^c^ *p <* 0.001: significant differences versus NTC. ^1^ *p <* 0.05; ^3^ *p <* 0.001: significant differences versus ASD. NTC: treated with distilled water (10 mL/kg); ASD: ASD animal treated with distilled water (10 mL/kg); ASD‐B: ASD animal treated at Bumetanide (4 mg/kg); ASD‐190, ASD‐760: ASD animals treated with the aqueous extract of *P. africanum* at doses of 190 and 760 mg/kg; NT‐190: neurotypical animal treated with the aqueous extract from *P. africanum* at dose of 190 mg/kg.

Compared with the NTC animals, there was a decrease in the number of neurons (*p <* 0.001; Mean: 26.46; SD: 15.12) in the negative control and an increase (*p <* 0.05; Mean: 49.69; SD: 15.12) in the positive control in the CA1 region. In the same area, compared with the negative control, an increase in neuron counting (*p <* 0.001) was observed in rats treated with 190 and 760 mg/kg extract in the pharmacological and positive control with respective Means of 43.77 (SD: 5.37), 38.81 (SD: 16.60), 48.63 SD: 11.94), and 49.69 (SD: 15.12). In the CA3 region, there was an increase (*p <* 0.001) in animals treated with 190 mg/kg compared with the negative control. In the dentate gyrus, there was a decrease in the number of neurons in the negative control compared to the neurotypical control (*p <* 0.05). The number of neurons in the amygdala compared to the NTC animals increased (*p <* 0.05) in animals treated with 760 mg/kg of the extract and decreased (*p <* 0.001) in the negatives. Compared with the negative control, there was an increase (*p <* 0.001) for the 190 and 760 mg/kg doses of extract, the positive control, and the pharmacological control (Figure 14).

## 4 Discussion

ASD is a neurodevelopmental condition affecting around one birth out of 150 worldwide. Atypical social communication, repetitive behaviors, and sensory‐motor issues characterize it. Pain reactivity is also typical in individuals with ASD (Elsabbagh et al. 2012; Bogdanova et al. 2022). The current study aimed to evaluate the anti‐stereotypic, anxiolytic, and analgesic potentials of the aqueous extract of *P. africanum* bark on a sodium valproate‐induced model of autism in rats. The oral administration of 800 mg/kg sodium valproate (VPA) to pregnant females on gestation days 11, 12, and 13 induced autistic‐like disorders in the offspring. Histone deacetylase (HDAC) is crucial during embryonic development, as it helps counteract the impacts of loss‐of‐function mutations in the growing embryo (Tseng et al. 2022). The present study shows that sodium valproate caused malformations in the offspring, the most representative of which were delayed eye opening and abdominal deformity. By hyperacetylating histones in the cells, VPA inhibited HDAC, resulting in the expression of anti‐apoptotic genes. This would lead to overexpression of genes from various exogenous and endogenous promoters, resulting in morphological defects (Phiel et al. 2001; Burenkova et al. 2015). The results obtained after exposure to VPA in the present study are similar to those of Zhao et al. (2019), who demonstrated that prenatal administration of sodium valproate during embryogenesis resulted in a polymalformative syndrome in the offspring (Zhao et al. 2019).

Behavioral analysis showed that VPA induced an anxious state and impaired social behavior in the offspring in the 3‐chamber sociability cage and the stereotypy test. Anxious behavior was also recorded in the open arena test. Indeed, anxiety disorders are the most characteristic behavioral disorders in autism (Kim et al. 2011; Raza et al. 2015). In line with this anxiety, the elevated plus maze test revealed a reduction in the time spent and the number of entries into the open arms while showing an increase in the same metrics for the closed arms. Additionally, there was a rise in grooming and sit‐up behaviors, along with a decrease in head drops. These observations are similar to those of Chaliha et al. 2020, who systematically demonstrated that gestational exposure to 600 mg/kg VPA in rodents at around 11.5 to 12.5 days gestation has disruptive effects on the three essential behavioral traits characteristic of ASD, with a reduction in social behaviors, an increase in repetitive behaviors and a rise in anxious behaviors reflecting cognitive rigidity (Chaliha et al. 2020). Indeed, histology shows that prenatal administration of sodium valproate in rodents resulted in lesions of the amygdala, cerebellum, and hippocampus, brain regions involved in regulating emotions and social behavior (Kim et al. 2011). In this way, VPA causes deficiencies in controlling emotions and behaviors. The development of anxiety in autism could be explained by an increase in GABA and serotonin in the brain. In the present study, analysis of markers of GABA metabolism showed a significant increase in GABA concentration, L‐GAD activity, and a substantial decrease in GABA‐T activity. Indeed, previous studies show that antagonists of H3R accelerate the liberation of 5‐HT and GABA (Witkin and Nelson 2004; Dai et al. 2007). However, exposure to valproic acid may affect the expression levels of histamine receptors (H2R and H3R) and inhibit their activities, potentially disrupting normal histamine‐mediated signaling pathways, negatively affecting social behavior and inducing stereotypy, two clinical aspects of the autism spectrum (Baronio et al. 2018). These increased levels of GABA, serotonin, and glutamate following exposure of rats in utero to valproic acid are in agreement with those of Bertelse et al. (2018) and Ay et al. (2024) in models of autism (Bertelse et al. 2018; Ay et al. 2024). Also, scientific studies and reviews indicate that autism is associated with high HDAC2 activity, making this enzyme a target for anti‐autistic agents (Raja et al. 2022). HDAC2 can induce anxiety by altering gene expression in the brain as HDAC (Peng et al. 2021; Morris et al. 2010; Whittle and Singewald, 2014). When over‐expressed, HDAC2 leads to a decrease in histone acetylation, thereby suppressing the transcription of genes essential to stress resistance and promoting the onset of anxiety, notably by down‐regulating the expression of neurotrophic factors in the hippocampus and amygdala (Peng et al. 2021; Morris et al. 2010; Whittle and Singewald, 2014). Furthermore, the pathogenesis of autism is characterized by an increase in the excitation/inhibition ratio in specific brain structures (Main and Kulesza 2017). During neuronal maturation, intracellular chloride ion concentration is elevated in immature neurons due to the high activity of the Na^+^‐K^+^‐Cl^−^ cotransporter (NKCC1). The GABA that opens the chloride‐ion‐permeable channels is then excitatory. In contrast, mature neurons have a low intracellular chloride ion concentration due to the high activity of the type 2 K‐Cl transporter (KCC2). Thus, our results corroborate those of Johannessen (2000) and Lin et al. (2013), who showed that sodium valproate administered to pregnant females increased GABA bioavailability in the offspring, either by increasing L‐GAD activity or by decreasing GABA‐T activity. This would result in cerebral hyperactivity, responsible for the anxiety state (Johannessen, 2000; Lin et al. 2013). Dufour‐Rainfray et al. (2010) have also shown that alteration of the serotonergic system in autism is responsible for the anxiety state in patients. This study showed increased cerebral tissue serotonin concentration compared with neurotypical animals. Indeed, depending on its receptors, serotonin or 5‐hydroxytryptamine (5‐HT) can be inhibitory or excitatory (Azmitia, Singh, Hou, et al. 2011). It has an excitatory role by binding to its 5‐HT2 and 5‐HT3 receptors and an inhibitory role by binding to its 5‐HT1 receptors. In autism, the number and function of 5‐HT1 receptors are reduced in favor of 5HT_2_ and 5HT_3_ receptors, thus the excitatory function. This increases the excitation/inhibition balance, characterizing an anxious state. These results are similar to those of Azmitia, Singh, Hou, et al. (2011), in which it is noted that administration of VPA to females at day 12.5 of gestation results in an increase in serotonin levels in the brains of the offspring (Azmitia, Singh, and Whitaker‐Azmitia 2011). The plant extract corrected the anxiety disorders associated with autism in this study, both biochemically, by reducing levels of the neurotransmitters involved in autistic conditions (GABA and 5‐HT) and behaviorally by reversing the direction of variation of the behavioral parameters assessed in the above‐mentioned anxiety state evaluation tests used in this work. This activity is strongly linked to the bioactive compounds identified by LCMS in the present study. In silico analysis revealed that the compounds identified (dimethoxy‐trihydroxy(iso)flavone isomer 1, 3,3′‐di‐O‐methyl ellagic acid, Methoxy‐tetrahydroxy(iso)flavone, β‐Sitostenone, and 5α‐Stigmast‐7,22‐dien‐3‐one) bind with high affinity, low energy, and good stability to histamine 3 receptors (H3R). This binding could activate these receptors by histaminergic agonism, thus overcoming the inhibitory action of valproic acid. This action could reduce glutamate and GABA levels, limiting their existing effects on autism and their anxiogenic power, as mentioned above (Johannessen 2000; Lin et al. 2013). In addition, the links of the compounds of the plant extract demonstrated in‐silico in this work at the HDAC2 could inhibit this enzyme and relieve autistic disorders associated with its hyperactivity, as aforementioned. Moreover, an aqueous extract of *P. africanum* would also interact with GABA metabolism, probably by the exact mechanism via some of its metabolites, such as tannins and flavonoids known to modulate synaptic transmission by inhibiting enzyme systems (Dlamini et al. 2019). In addition, tannins and polyphenols could modulate L‐GAD and GABA‐T activity (Johannessen 2000). Thanks to their anxiolytic properties, flavonoids modulate serotonin activity at their receptors, lowering its concentration in the brain (Okponanabofa et al. 2019).

Anxiety in autism is also thought to be due to oxidative stress. Prenatal administration of sodium valproate on gestation days 11, 12, and 13 resulted in oxidative stress in the offspring, with increased MDA concentrations, decreased SOD activity, and reduced glutathione (GSH) concentrations in the cerebellum, hippocampus, amygdala, and prefrontal cortex. These results are similar to those of Chen et al. (2021), who showed that VPA increased brain and blood MDA concentrations, SOD activity, and GSH concentration. VPA is believed to cause hypermethylation of antioxidant enzymes (GSH and SOD) in the brains of autistic children (Narita et al. 2010). This hypermethylation prevents the expression of genes that regulate the synthesis of antioxidant enzymes (Narita et al. 2010). Indeed, GSH plays an essential role in the elimination of mitochondrial ROS. Therefore, a drop in GSH would increase ROS, resulting in oxidative stress responsible for inflammation in the brains of people with ASD (Liu et al. 2022). This inflammation is thought to be accountable for ASD behavior. In addition, the increased vulnerability to oxidative stress observed in autism is linked to decreased glutathione levels and is specific to the brain region. The cerebellum and temporal cortex of children with autism show more significant differences in glutathione concentrations compared with controls. The cerebellum plays a vital role in motor control and cognitive functions, such as attention and language. The temporal cortex involves social perception, joint attention, and expressive language. Increased damage to these regions due to low glutathione redox status could explain certain behavioral traits (Castejon and Spaw 2014). The oxidative stress thus present in ASD is responsible for cellular damage, which would explain the loss and decrease in the number of neurons observed in the present study's cerebellum, amygdala, and hippocampus. Indeed, it is well‐recognized that hyperexpression of HDAC2 in the autism spectrum can contribute to neuronal death (Guan et al. 2009). Although HDAC2 is diffusely distributed in the cerebrum, the most densely stained neurons are located in the CA1‐CA3 regions of the hippocampus (Yao et al. 2012; Sun et al. 2019). Higher levels of HDAC2 lead to tighter DNA packaging, restricting access to genes essential for neuronal development and function (Tseng et al. 2022). The cell's capacity to fight reactive oxygen species (ROS) is diminished when HDAC2 suppresses the production of antioxidant genes through its histone deacetylase activity. The regulation of transcription factors like Nrf2, which is crucial to the antioxidant defense system, is commonly a part of this mechanism (Wang et al. 2012; Peng et al. 2015). The aqueous extract of *P. africanum* reduced oxidative stress in the brain, suggesting the plant extract has antioxidant activity. Qualitative phytochemical analysis of the extract showed the presence of tannins, phenols, and flavonoids whose cytoprotective and neuroprotective properties are strongly correlated with their antioxidant properties as free radical scavengers (Dlamini et al. 2019). The inhibitory action of the plant extract compounds on HDAC2, as mentioned in silico, would be a probable antioxidant pathway to limit oxidative neuronal damage linked to the autism spectrum.

People with ASD often have sensory abnormalities. Sensory hyper‐ or hypo‐reactivity is frequently observed in people with autism. Sodium valproate in this study caused nociceptive hypersensitivity on the hot plate. These observations are similar to those of Yumi and collaborators, who were able to demonstrate peripheral hyperalgesia in young people and adults with autism. Indeed, the increase in serotonin and GABA concentration would result in algesia, leading to inflammation that causes a pronociceptive effect (Umesawa et al. 2020). This would result in an imbalance in the excitation/inhibition balance in the somatosensory cortex or amygdala. These modifications could alter the processes of integration and encoding of nociceptive stimuli and thus disrupt the elaboration of the sensation of pain (Lévesque et al. 2011). Aqueous extract of *P. africanum* bark increased nociceptive response latency, thereby reducing hypersensitivity. The chemical compounds present in the extract enabled this sensitivity modulation. Specifically, tannins and glucosides, due to their anti‐inflammatory, antipyretic, and analgesic properties (Okponanabofa et al. 2019), are capable of inhibiting the inflammation caused by high serotonin levels, leading to a reduction in pronociceptive effects in the somatosensory cortex and consequently a decline in hypersensitivity. The decline of hypersensitivity is due to thalamocortical dysconnectivity since the thalamus is a critical functional hub for relaying sensory information to the cortex and modulating motor signals (Horata et al. 2024). Further studies are needed to investigate the effects of *P. africanum* on these neuronal circuits.

## 5 . Conclusion

The present study aimed to evaluate the effects of aqueous extract of *P. africanum* bark on anxiety and pain sensitivity in a sodium valproate‐induced model of autism in young Wistar rats. Administration of 800 mg/kg sodium valproate orally on days 11, 12, and 13 induced social interaction deficits, stereotypies, anxiety, pain hypersensitivity, altered GABAergic and serotonergic neurotransmission, and oxidative stress in the offspring. At the end of the treatment, the aqueous extract of *P. africanum* first attenuated the anxious behavior of the animals and their hypersensitivity to pain induced by sodium valproate by restoring GABAergic and serotoninergic transmission. Second, the extract significantly increased the reduced glutathione and superoxide dismutase activity concentration and decreased malondialdehyde concentration in rats' cerebellum, prefrontal cortex, hippocampus, and amygdala. Third, the aqueous extract of *P. africanum* improved the alterations observed in this study's various brain areas of interest. Molecular docking studies demonstrated that *P. africanum* compounds can interact with HDAC2 and H3R in the autism spectrum. These observations suggest that *P. africanum* has antioxidant, anxiolytic, and analgesic properties mediated by its neuromodulatory activities. Although further investigations are required, these results show that the aqueous extract of *P. africanum* bark can be used in traditional medicine to treat certain neurological disorders.

## Author Contributions

**Ambani Omgba Jeanne Julie**: investigation, methodology, formal analysis, writing – original draft. **Ngouateu Omer Bébé**: conceptualization, validation. **Mengue Ngadena Yolande Sandrine**: conceptualization, writing – review and editing, data curation. **Owona Pascal Emmanuel**: investigation, visualization, writing – review and editing. **Kandeda Kavaye Antoine**: methodology, data curation, formal analysis. **Ambamba Akamba Bruno Dupon**: methodology, data curation. **Nongni Piebeng Quentin Cicilien**: methodology, software. **Ngang Nguema Franck Emmanuel**: visualization, formal analysis. **Ngondi Judith Laure**: validation. **Bilanda Danielle Claude**: conceptualization, data curation, writing – review and editing. **Dzeufiet Djomeni Paul Désiré**: conceptualization, project administration, supervision.

## Conflicts of Interest

The authors declare no conflicts of interest.

### Peer Review

The peer review history for this article is available at https://publons.com/publon/10.1002/brb3.70408.

## Data Availability

The data that support the findings of this study are available from the corresponding author upon reasonable request.
